# Kallfu and Wenutram: two Chilean flaxseed varieties with contrasting mucilage production, composition, and structure

**DOI:** 10.3389/fpls.2025.1626044

**Published:** 2025-08-28

**Authors:** Susana Grant-Grant, Dayan Sanhueza, Pablo Sepúlveda-Orellana, Sebastián Zúñiga-Pozo, J. Sebastián Contreras-Riquelme, José M. Alvarez, Asier Largo-Gosens, Susana Saez-Aguayo

**Affiliations:** ^1^ Centro de Biotecnología Vegetal, Laboratorio Mucilab, Facultad de Ciencias de la Vida, Universidad Andrés Bello, Santiago, Chile; ^2^ Agencia Nacional de Investigación y Desarrollo (ANID) - Anillo de Investigación en Ciencia y Tecnología - Chilean Fruits Cell Wall Components as Biotechnological Resources (CHICOBIO) ACT210025, Talca, Chile; ^3^ ANID Millenium Science Initiative Program, Millennium Institute for Integrative Biology (iBio), Santiago, Chile; ^4^ ANID - Millennium Nucleus in Data Science for Plant Resilience (PhytoLearning), Santiago, Chile; ^5^ Área de Fisiología Vegetal, Departamento de Ingeniería y Ciencias Agrarias, Universidad de León, León, Spain

**Keywords:** seed mucilage, flaxseed mucilage, mucilage structure, Rhamnogalacturonan-I, Rhamnogalacturonan-II, cell wall proteins, flaxseed mucilage proteomic

## Abstract

Seed mucilage, rich in complex polysaccharides, serves diverse functions upon hydration, including soil adhesion, dispersal, and stress protection, making it valuable for food and pharmaceutical applications. Its water-holding capacity aids in food moisture retention, while its emulsifying properties enable various culinary and pharmaceutical uses. Mucilage from flax seeds also offers potential as a bioencapsulation material, with studies exploring its role in drug and probiotic delivery systems targeting the gastrointestinal tract. To investigate differences in mucilage characteristics, we compared two Chilean flaxseed cultivars, Kallfu and Wenutram, which differ in mucilage content. A combination of biochemical, cytological, and proteomic analyses was used to assess composition and structure. Our analyses revealed that flaxseed mucilage (FM) is predominantly composed hemicellulose (HC) and branched rhamnogalacturonan I (RG-I), with variations in RG-I branching patterns observed between cultivars. Minor constituents, such as homogalacturonan (HG) and rhamnogalacturonan II (RG-II), also contribute to mucilage architecture. Proteomic analysis identified a diverse set of proteins in FM, some of which may be involved in mucilage modification. Differences in mucilage content and composition between Kallfu and Wenutram highlight the structural complexity of FM and its potential functional implications. These findings provide new insights into how variations in FM composition influence its architecture and release properties, advancing the understanding of cell wall structure in relation to mucilage extrusion.

## Introduction

Seed mucilage is a gel-like structure synthesized by seed coat epidermal cells, enriched in complex polysaccharides, such as pectins, hemicelluloses, and cellulose (Phan and Burton, 2018; Tsai et al., 2017). When seeds are imbibed with water, these polysaccharides form a hydrogel that serves multiple functions including adhesion to moist soils, dispersal by animals, protection against abiotic and biotic stresses, and water retention to aid in germination (Phan and Burton, 2018). The water-holding capacity of mucilage, attributed to its hydroxyl groups and proteins, enhances the absorption and retention of water in food and industrial products (Alpizar-Reyes et al., 2017; Naveed et al., 2019; Tosif et al., 2021). Among its many attributes, mucilage demonstrates emulsifying properties for particle suspension, thickening, encapsulation, and film coating (Bustamante et al., 2017; Kučka et al., 2022; Tosif et al., 2021). Additionally, plant seeds mucilage shows antioxidant potential, mainly due to the presence of phenolic compounds within the polysaccharide matrix (Tosif et al., 2021; da Silveira et al., 2024). These versatile characteristics make mucilage an attractive option for the bioencapsulation of biological molecules, particularly proteins. Several plant sources, including chia (*Salvia hispanica*), okra (*Abelmoschus esculentus*), tamarind (*Tamarindus indica*), flax (*Linum usitatissimum*), *Plantago mayor* and cress seeds (*Lepidium sativum*), have been investigated for their mucilage content and potential applications in the food and pharmaceutical sectors (Francoz et al., 2018; Tosif et al., 2021, Tsai et al., 2017).

Flax (Linum usitatissimum) is an economically significant annual herbaceous plant with a history dating back to ancient Egypt (Ehrensing, 2008). It is an excellent candidate for mucilage-related studies due to its ability to accumulate substantial amounts of polysaccharides (Soto-Cerda et al., 2018; Puligundla and Lim, 2022; Troshchynska et al., 2022). Flaxseed is also rich in oil, particularly alpha-linolenic acid, which is recognized for its beneficial effects on human health (Singh et al., 2011; Bernacchia et al., 2014; Nowak and Jeziorek, 2023). The mucilage content of flaxseed is approximately 7-10% of its dry weight, a level comparable to chia seeds (6-8%) but lower than that of *Plantago major* seeds, which exhibit a mucilage content of 15% (Fedeniuk and Biliaderis, 1994; Tosif et al., 2021). Furthermore, flaxseed mucilage (FM) has demonstrated potential as a delivery system for drugs and probiotics targeting the mucosa and gastrointestinal system (Fedeniuk and Biliaderis, 1994; Bouaziz et al., 2016; Devi and Bhatia, 2019; Nerkar and Gattani, 2011; Rashid et al., 2019; Sheikh et al., 2020).

Flaxseed mucilage exhibits an organized layered structure and undergoes a peeling-like release mechanism (Miart et al., 2019). This release mechanism is distinct from that of *Arabidopsis thaliana*, which forms a volcano-like shape structure, called columella, surrounded by a donut-shaped mucilage that rapidly releases upon contact with water (Western et al., 2000; Macquet et al., 2007; Voiniciuc et al., 2015; Saez-Aguayo et al., 2013). Given this difference, it is expected that the organization of mucilage is not the only distinction between Arabidopsis and flax. Despite these differences, both FM and Arabidopsis mucilage are predominantly composed of pectins, with rhamnogalacturonan-I (RG-I) being the main structural component (Macquet et al., 2007). However, unlike in Arabidopsis, the FM RG-I domain is interrupted by short chains of homorhamnan (HR) and homogalacturonan (HG) (Qian et al., 2012). In contrast, the RG-I backbone in Arabidopsis mucilage is largely unbranched, whereas FM exhibits branches that contain arabinose, galactose, and fucose (Fedeniuk and Biliaderis, 1994; Macquet et al., 2007; Elboutachfaiti et al., 2017; Ding et al., 2018). These branches typically bind to the O-3 position of rhamnose, a unique characteristic of FM (Naran et al., 2008; Qian et al., 2012; Elboutachfaiti et al., 2017). Furthermore, FM contains hemicellulose, mainly composed of arabinoxylans with side chains containing galactose and fucose (Naran et al., 2008; Ding et al., 2018).

There are approximately 48,000 accessions of flax in different genebanks, with a core collection of 300 flaxseed cultivars and 92 fiber types consistently cultivated (Cloutier et al., 2009). In this study, we examined FM of two cultivars, Kallfu and Wenutram, developed in Chile by the Centro de Genómica Nutricional Agroacuícola (CGNA, Temuco, Chile). Both Kallfu and Wenutram were developed for food, oil production, and animal feed (Centro de Genómica Nutricional Agroacuícola, 2017). However, they differ significantly in mucilage content, with Wenutram having lower mucilage content than Kallfu. The contrasting mucilage content of these cultivars makes it particularly interesting to analyze whether differences in mucilage content also affect its structure. Using a combination of histological, biochemical, and immunodetection techniques, we identified significant differences in the composition and structure of RG-I, rhamnogalacturonan-II (RG-II) content, methyl ester content, and hemicellulose domains between the two cultivars. Additionally, given the high protein content in FM, we successfully identified and quantified FM proteins for the first time, enhancing our understanding of the FM composition and differences between the two cultivars.

This work contributes to a deeper understanding of FM composition and how variations in different components of FM can influence its behavior and structure. A better understanding of FM is valuable for developing new applications in various technological fields, such as oil production, recombinant protein production, and the development of new biopolymers.

## Materials and methods

### Plant material

The Kallfu and Wenutram flax seeds were developed and kindly provided by the Centro de Genómica Nutricional Agroacuícola (CGNA), Temuco, Chile (https://www.cgna.cl).

### Extrusion analysis

Mature seeds were placed on a 0.5% agarose gel containing 0.002% or 0.02% Ruthenium Red, with or without 50 mM or 50 µM EDTA. The seeds were incubated overnight at room temperature (RT) to observe mucilage release. Images were captured using the Leica stereo zoom S9I microscope. The size of the extrusion halo was determined by analyzing the images using ImageJ software. Statistical analysis was performed using a t-test with a significance level set at *p* < 0.0005.

### Release of flax seed mucilage and protein content

Five grams of dried flax seeds were soaked in 50 mL of MilliQ water with continuous agitation for 16 hours. The soluble mucilage was obtained by filtering the supernatants through Miracloth, which were later lyophilized, weighed, and stored at room temperature. All experiments were conducted using the soluble mucilage.

Protein quantification of soluble mucilage was performed using the BCA assay (Pierce™ BCA Protein Assay Kit, Thermo Fisher Scientific) following the manufacturer’s instructions. Briefly, 2 mg of freeze-dried mucilage were weighed and dissolved in 300 µL of Milli-Q water. An aliquot of 20 µL from this solution was used for protein quantification.

### Flax seed mucilage AIR preparation, pectin- and hemicellulose-enriched fraction isolation, acid hydrolysis, and sugar analysis

The freeze-dried soluble mucilage was ground using liquid nitrogen and then incubated with 80% ethanol for 2 hours at room temperature then the samples were centrifuge at 6,000 rpm for 10 minutes, the supernatants were discarded, the pellet is resuspended in 80% ethanol and incubated overnight. Lipids were removed by incubating the samples with methanol:chloroform (1:1 v/v) two times for 2 hours each, followed by two washes with acetone for 1 hour each. The resulting residue, known as alcohol insoluble residues (AIR), was air-dried overnight at room temperature (RT).

To obtain enriched fractions of pectin (EPF) and hemicellulose (EHF), 60-100 mg of AIR was incubated with 4 mL of 0.5 M imidazole pH 7 with constant agitation at RT. The samples were centrifuged at 4,000 rpm for 10 minutes, and the supernatant was stored at -20°C. The pellet was incubated again with imidazole, and the process was repeated until the supernatant became clear. The pellet was then incubated with 0.2 M ammonium oxalate pH 4.3 overnight at 60°C. After centrifugation at 4,000 rpm for 10 minutes, the supernatant was recovered and stored at -20°C. The enriched pectin supernatants were dialyzed using a 10,000 MWCO membrane and then freeze-dried. The pellet was washed once with water and incubated with 6 M NaOH and 1% sodium borohydride (NaBH_4_) under constant agitation at 37°C overnight. The supernatant was collected, and a second incubation was performed for 24 hours. After centrifugation at 4,000 rpm for 10 minutes, the supernatant containing hemicellulose was collected, dialyzed, and freeze-dried.

For acid hydrolysis and sugar analysis, 1-2 mg of AIR, EPF and EHF, were hydrolyzed with 400 µL of 2 M trifluoroacetic acid (TFA) at 121°C for 40 minutes using allose and myo-inositol (250 µM each) as internal standards. The TFA was evaporated at 45°C under a flow of nitrogen gas. The samples were washed twice with 400 µL of 100% isopropanol and dried with nitrogen at 45°C. The hydrolyzed products were resuspended in 600 µL of MilliQ water, sonicated for 15 minutes, filtered through a syringe filter (pore size: 0.22 µm), and transferred to a vial to be analyzed by HPAEC-PAD (Dionex ICS3000) as described by Saez-Aguayo et al., 2021 and Sanhueza et al., 2024.

### Methyl ester quantification

Fifty microliters of a solution containing either 5 µg/µL AIR or 3 µg/µL of EPF solution, were saponified with 50 µL of 1 M NaOH at 4 °C on ice for 1 hour. The reactions were stopped by neutralizing with 50 µL of 1 M HCl, and the volume was brought to 300 µL with water, an extra dilution was performed for AIR, to the final 300 µL, 1200 µL of extra water was included. From the saponified solution, 50 µL were mixed with 100 µL of 200 mM Tris-HCl pH 7.5, 40 µL of 3-methyl-2-benzothiazolinone hydrazone (MBTH, 3 mg/mL), and 20 µL of alcohol oxidase (0.02 U/µL) from *Pichia pastoris* (Sigma). The mixture was incubated for 20 minutes at 30°C.

After incubation, 200 µL of a solution containing 0.5% w/v sulfamic acid (H_3_NSO_3_) and 0.5% w/v ferric ammonium sulfate (NH_4_Fe(SO_4_)_2_ * 12H_2_O) was added to each sample, and incubation continued for another 20 minutes at RT. Finally, the samples were diluted with 600 µL of water, and the absorbance was measured at 620 nm. A standard curve from 0 to 10 µg/µL was used to determined methyl ester content.

### RG-II detection by electrophoresis

To analyze RG-II, the samples were processed as described by Chormova et al. (2014). Two milligrams of freeze-dried AIR were resuspended in 100 µL of digestion buffer (0.01% driselase (*Basidiomycetes* sp., Sigma-Aldrich), 50 mM sodium acetate, pH 4.8) and incubated overnight at 37°C. Then, 8 µL of the digestion mixture was loaded onto a 26.4% acrylamide gel. As a loading control, 6 µL of RG-II dimer (1.8 µg/µL) and RG-II monomer (1.8 µg/µL), prepared from Arabidopsis, were also loaded onto the gel. Then electrophoresis and gel staining were perform as described in Chormova et al., 2014. A relative quantification of pixel intensity was performed using a scanned image of the RG-II electrophoresis gel and the software ImageJ version 1.54p. The image was first converted to 8-bit format and subsequently color-inverted. Same-size areas were then defined and applied uniformly to all bands present in the image, from which the pixel intensity values were recorded. For background signal correction, adjacent background regions were measured for each band and their values were subtracted from the original band intensity. The resulting values are expressed as area-normalized intensities, based on an arbitrary grayscale scale ranging from 0 to 255.

### RG-I isolation and cell wall domain detection

Fractions enriched in different domain were obtained by size exclusion chromatography (SEC) of the endopolygalacturonase-treated isolated pectin as described in Sanhueza et al., 2024. First, 9 µg of EPF was saponified by overnight incubation with 0.5 M Na_2_CO_3_ at 4°C, then neutralized with acetic acid. The saponified EPF was precipitated with 2 volumes of ethanol, centrifuged for 2 minutes at maximum speed, and rinsed twice with 80% ethanol. Next, the saponified EPF was digested overnight at 20°C with 2.25 U/mL of endopolygalacturonase (*Aspergillus aculeatus*, Megazyme) in Pyridine: Acetic acid:Water (PyAW 1:10:200) solution. The digestion mixture was loaded onto a BioGel P-30 column with a fractionation range of 2,500 to 40,000 MW (2.5 x 57 cm) and eluted with PyAW 1:1:98 at 1 mL/min. To detect the fractions where the different domains eluted, 20 µL of each collected fraction was used to quantify total uronic acids (Blumenkrantz and Asboe-Hansen, 1973) and total neutral sugar content (Schonenberger, 1957). The enriched fractions were selected based on the first quantification peak. Using this peak as a reference, three preceding and five subsequent fractions were collected, resulting in nine total fractions analyzed. Fractions enriched in RG-I were dried using a speed vacuum.

### Dot blot assays and signal quantification

To detect different polysaccharide domains, serial dilutions of AIR (20 µg/µL), EPF (3 µg/µL), EHF (3 µg/µL) and RG-I isolated domain (200 ng/µL) were spotted onto nitrocellulose membrane. The membrane was then blocked for 1 hour at RT with constant agitation with 2% skimmed milk in TBS-T (1x TBS (50 mM Tris-Cl, pH 7.5, 150 mM NaCl) with 0.1% Tween-20). Primary antibodies, diluted 1:50, were incubated with agitation for 2 hours at RT in 1% skimmed milk in TBS-T. The membrane was rinsed three times with TBS-T, each rinse including 5 minutes of agitation. Alkaline phosphatase (AP)-conjugated secondary antibodies, diluted 1:2,000 in 1% skimmed milk in TBS-T, were incubated for 1 hour at RT. The membrane was rinsed as previously described. Finally, the membrane was incubated with 1-STEP NBT/BCIP solution for colorimetric detection of alkaline phosphatase activity. Dot blots were scanned, and the area and mean intensity of each dot were analyzed using ImageJ. Average values for each dilution were summed and plotted in a heatmap (see **Supplementary Figure 1**).

### Histological sections and immunodetection

The seeds were cut into three fragments to facilitate penetration of the fixation buffer (4% paraformaldehyde, 0.1 M sodium phosphate buffer pH 7.4, 1% w/v saccharose, and 0.05% Tween 20). The fragments were incubated in the fixation buffer for 1 hour under vacuum to 60 KPa, after which the buffer was replaced with fresh solution and the samples were incubated overnight at 4°C. Dehydration was carried out by incubating the samples in increasing concentrations of ethanol (30%, 50%, 70%, 80%, 90%), with each incubation lasting 1 hour at 4°C. Following a 1-hour incubation in 100% ethanol at 4°C, the samples were placed under vacuum for the first 30 minutes. A second incubation in 100% ethanol was performed overnight at 4°C. Next, the samples were incubated three times for 1 hour each with increasing concentrations of LR White Resin (1:2, 1:1, 2:1, resin:ethanol) at 4°C, followed by two incubations in 100% resin, with the first 30 minutes under vacuum. Finally, the samples were placed in molds and incubated at 60°C for 48 hours to form blocks for sectioning.

Sections were prepared using a Leica EM UC7 ultramicrotome, producing 1.5 µm sections. These sections were pre-treated with 100% alcohol and dried on a hot plate to enhance staining penetration. Toluidine blue staining was performed using a 1% toluidine blue solution in 0.1 M sodium phosphate buffer (pH 7.4). The samples were incubated for 5 minutes at RT and then washed with distilled water.

For immunolabeling, the sections were first blocked with 2% skimmed milk in 1x TBS for 30 minutes at RT. The primary antibody, diluted 1:10, was then incubated in 1% skimmed milk in 1x TBS with 0.05% Tween for 2 hours at RT. After incubation, the sections were washed three times with 1x TBS for 5 minutes each. The secondary antibody, diluted 1:500, was incubated in 1% skimmed milk in 1x TBS with 0.05% Tween for 1.5 hours at RT. Primary antibodies were obtained from Kerafast, and Alexa488 Goat anti-mouse or anti-rat were obtained from Thermos Fisher Scientific.

Arabinofuranosidase digestion was performed on the sections using 50 U/mL α-L-Arabinofuranosidase (Megazyme) in 100 mM sodium acetate buffer pH 4.4, incubated for 1 hour at 37°C. The digestion was stopped by rinsing with MilliQ water. Immunolabeling was performed as described previously.

The labeled sections were analyzed using a Leica SP8 confocal microscope, and images were processed with ImageJ software.

### Protein analysis

Protein isolation from mucilage was carried out following the method described by Tsai et al. (2017). Briefly, nine seeds from each cultivar were soaked overnight in MilliQ water. The extracted mucilage was collected and freeze-dried. The mucilage was then deglycosylated using 270 µL of trifluoromethanesulfonic acid (Sigma-Aldrich) and 30 µL of anisole (Sigma-Aldrich) at 4°C for 2 hours. The solution was neutralized with pyridine (Sigma-Aldrich) and dialyzed overnight at 4°C using a 3,500 MWCO membrane in MilliQ water. The dialyzed solutions were freeze-dried, and the resulting proteins were resuspended in 1x SDS loading buffer (50 mM Tris-Cl (pH 6.8), 2% (w/v) SDS, 0.1% (w/v) bromophenol blue, 10% (v/v) glycerol, 100 mM DTT). The proteins were separated by SDS-PAGE, and after electrophoresis, the gel was stained with Coomassie blue. Protein bands were excised and sequenced at the Proteomics Core Facility at Michigan State University.

### Proteolytic digestion

Gel bands were digested in-gel according to Shevchenko et al. (2006), with modifications. The gel bands were first dehydrated with 100% acetonitrile and then incubated with 10 mM dithiothreitol in 100 mM ammonium bicarbonate (pH ~8) at 56°C for 45 minutes. After dehydration, the gel bands were incubated in darkness with 50 mM iodoacetamide in 100 mM ammonium bicarbonate for 20 minutes. They were then washed with ammonium bicarbonate and dehydrated again. Sequencing-grade modified trypsin was prepared at a concentration of 5 ng/µL in 50 mM ammonium bicarbonate, and approximately 100 µL of this trypsin solution was added to each gel band, ensuring that the gel was completely submerged. The gel bands were then incubated overnight at 37°C. Peptides were extracted from the gel by sonication in a solution of 60% acetonitrile/1% trifluoroacetic acid and vacuum dried to approximately 2 µL.

### LC/MS/MS analysis

Peptide samples were resuspended in 20 µL of 2% acetonitrile/0.1% trifluoroacetic acid. A 5 µL injection was automatically loaded onto a Thermo EASYnLC 1200 system equipped with a Thermo Acclaim PepMap RSLC C18 trapping column (0.1 mm x 20 mm). Bound peptides were washed for approximately 5 minutes with buffer A (0.1% formic acid). Subsequently, the peptides were eluted over 35 minutes onto a Thermo Acclaim PepMap RSLC resolving column (0.075 mm x 500 mm) using a gradient of buffer B (0.1% formic acid/80% acetonitrile), progressing from 8% to 40% over 24 minutes, followed by ramping to 90% B for 25 minutes. The column temperature was maintained at 50°C using an integrated column oven (PRSO-V2, Sonation GmbH, Biberach, Germany).

Eluted peptides were sprayed into a Thermo Scientific Q-Exactive HF-X mass spectrometer via a FlexSpray spray ion source. Survey scans were performed in the Orbitrap at a resolution of 60,000 (determined at m/z), and the top 15 ions from each scan were subjected to automatic higher-energy collision-induced dissociation (HCD), with fragment spectra acquired at a resolution of 15,000. The resulting MS/MS spectra were converted to peak lists using MaxQuant 1.6.3.4 and searched against all available *Linum usitatissimum* protein sequences from JGI (downloaded from Phytozome 13 (https://phytozome-next.jgi.doe.gov/), v1.0). Common laboratory contaminants were included from the Generalized Proteomics data Meta-analysis (https://www.thegpm.com). The Andromeda search algorithm was used for the search of proteins identifications. The Andromeda output was then analyzed using Scaffold 5.2.2 (Searle, 2010) to probabilistically validate protein identifications. Proteins were considered valid if they passed the 1% FDR confidence filter set in Scaffold.

Additionally, an exclusive unique peptide counts of 2 was established, meaning each identified protein had to have at least two unique and exclusive peptides. A peptide threshold of 1% FDR was also applied to ensure high confidence in identification. This selection provides us with several groups based on the probability of the protein being present. As the proteins within each group have a variable probability of being present, the protein with the highest probability according to Scaffold 5 was chosen. The data was normalized to enable effective comparisons, aiming for similar distributions among replicates. Different normalization methods, including min-max normalization, normalization by the average of each replicate, and DESeq2 normalization (Love et al., 2014), were evaluated.

In parallel, Gene Ontology (GO) terms were assigned to the flax proteome using InterProScan (Jones et al., 2014), integrated into the Blast2GO suite. This GO annotation was used to filter the protein groups, removing those associated with seed functions (**Supplementary Table 1**). Enriched GO terms were identified using GOATOOLS, and histograms were generated to visualize data distribution (**Supplementary Figures 2**, **3**).

To understand the biological processes involving these proteins, those found in all samples of at least one variety were selected. These proteins were then categorized by second level GO terms (**Supplementary Figure 1**).

To identified differentially quantified proteins, a Mann-Whitney U test was employed. Although the proteins displayed a distribution resembling a negative binomial distribution, the Mann-Whitney U test was chosen to avoid assumptions about distribution patterns. An adjusted p-value <0.1, using the Benjamini-Hochberg procedure, was applied as the selection criterion.

## Results

### The Kallfu cultivar has a larger extrusion halo, bigger mucilage secretory cells, and higher mucilage content compared to Wenutram

**Figure 1:**
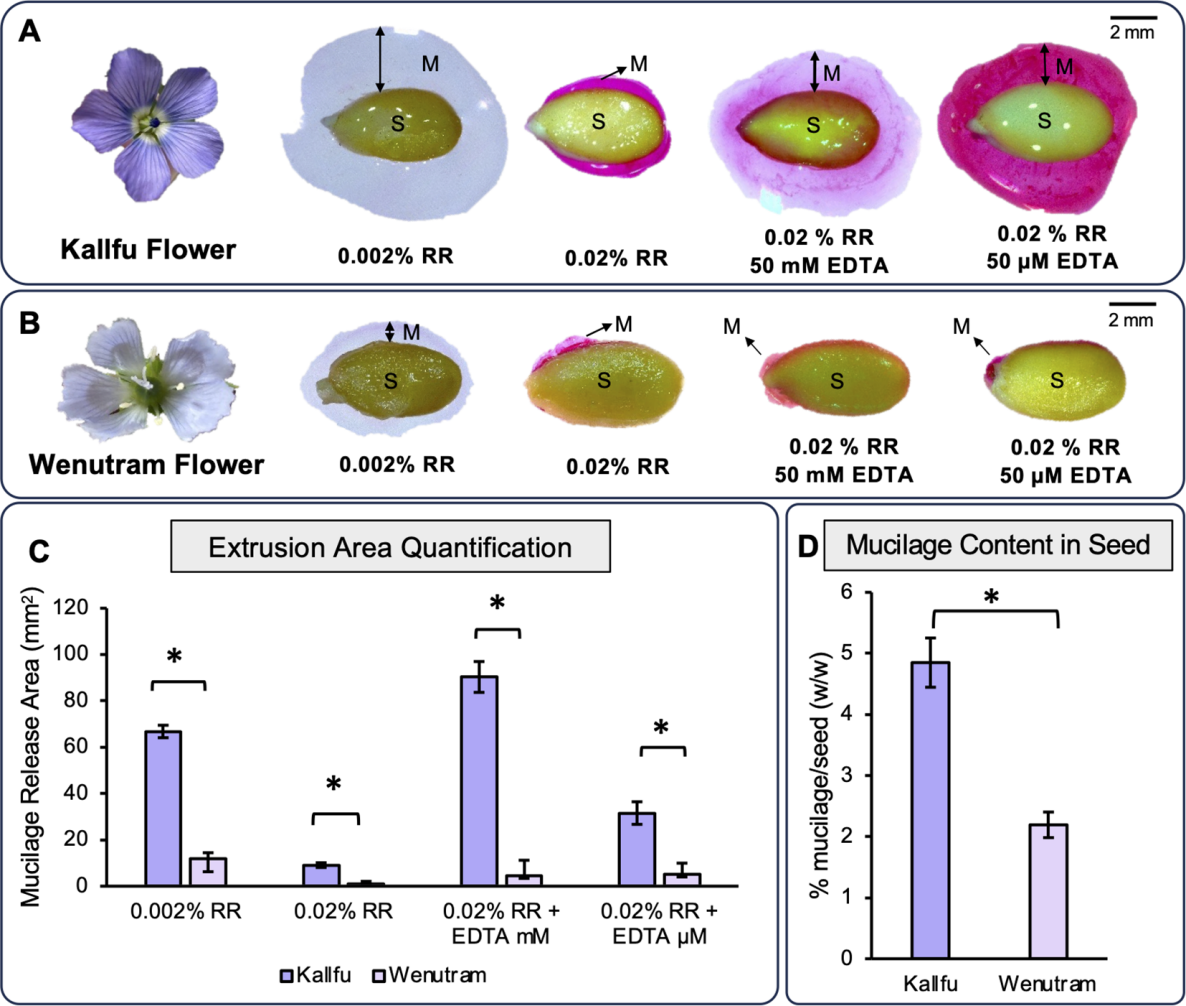
Profile of mucilage extrusion in Kallfu and Wenutram seeds: **(A, B)** Mucilage release from both Chilean flax seed varieties. These panels depict the flowers of Kallfu and Wenutram, along with their mucilage extrusion profiles under two different concentrations of EDTA (50 mM and 50 µM) and, in the presence of Ruthenium Red (RR). The images highlight notable differences between the two varieties, particularly the contrasting mucilage release behavior, with Wenutram exhibiting minimal to no mucilage extrusion. Bar = 2 mm. **(C)** Quantification of mucilage extrusion areas in both Chilean flax seed varieties. The graph shows the quantification and comparison of the mucilage extrusion area in Kallfu and Wenutram under different RR and EDTA conditions. **(D)** Analysis of dried mucilage weight distribution in both Chilean flax seed varieties. The graph shows the percentage of seed weight attributed to mucilage released upon water imbibition. Mucilage weight was measured after drying. Statistical analysis was performed using a t-test, with a significance threshold of p-value < 0.0005. * symbol indicates statistical differences.

To compare mucilage release between the two cultivars, seeds were stained with Ruthenium red (RR), which binds negatively charged molecules such as galacturonic acid in pectin backbones (Western et al., 2000). Two RR concentrations (0.002% and 0.02% w/v) were tested, along with two EDTA treatments (50 mM and 50 μM) to assess the effect of Ca²^+^ chelation. The diluted RR revealed clear extrusion halos in both cultivars, while the concentrated RR produced stronger staining but smaller halos, likely due to ionic strength (Roulard et al., 2016) (**Figures 1A–C**). In both conditions, Kallfu showed a larger extrusion halo than Wenutram. EDTA, which disrupts HG “egg-box” structures by chelating Ca²^+^ (McFarlane et al., 2014), also resulted in larger halos in Kallfu under both concentrations. Notably, lower EDTA levels enhanced coloration in Kallfu (**Figures 1A–C**). Soluble mucilage content was then quantified by freeze-drying and comparing dry weights, revealing that Kallfu produces twice as much mucilage per gram of seed as Wenutram (**Figure 1D**).

To evaluate whether differences in mucilage release were due solely to content or also structure, seed coat anatomy was examined. Histological sections stained with toluidine blue showed that Kallfu has larger mucilage secretory cells (MSCs) than Wenutram (**Supplementary Figure 4**). In both cultivars, MSCs stained purple—a color indicative of polysaccharides (O'Brien, 1964)—supporting the conclusion that Kallfu accumulates more mucilage in MSCs.

### Kallfu and Wenutram mucilage differ in their monosaccharide composition

The monosaccharide profiles of Kallfu and Wenutram mucilage were analyzed to identify structural differences (**Table 1**). Kallfu showed higher levels of most monosaccharides, except glucose, which was more abundant in Wenutram. Overall, Kallfu had nearly double the total monosaccharide content compared to Wenutram. Xylose was the most abundant sugar in both cultivars, accounting for 33% in Kallfu and 29% in Wenutram, followed by galactose in Kallfu (17%) and glucose in Wenutram (16%) (**Table 1**). To further investigate cultivar differences, pectin- and hemicellulose-enriched fractions were extracted from AIR. In the EPF, Kallfu was enriched in xylose (26%), rhamnose (23%), galactose (16%), and arabinose (12%), while Wenutram contained xylose (23%), rhamnose (20%), glucose (16%), galactose (15%), and arabinose (13%) (**Table 1**). The elevated levels of rhamnose, galactose, and arabinose suggest the presence of highly branched RG-I. The high xylose content indicates contributions from other polymers such as xylan, xyloglucan, and arabinoxylan. Molar ratio analysis in the EPF further highlighted cultivar differences. The Rha/GalA ratio revealed that rhamnose levels were approximately twice those of galacturonic acid, with Wenutram exhibiting a slightly higher ratio (2.51) than Kallfu (2.46). These values suggest that the flax mucilage is predominantly composed of rhamnogalacturonan-I (RG-I), with a probable contribution of homorhamnan, which warrants further structural characterization (**Supplementary Table 2**).

**Table 1:** Monosaccharide composition of AIR, EPF and EHF from FM.

AIR			EPF			EHF		
Sugar	Kallfu	Wenutram	Sugar	Kallfu	Wenutram	Sugar	Kallfu	Wenutram
Fucose	34.8 (0.8)	13.6 (1.3)	Fucose	50.2 (0.6)	31.5 (0.9)	Arabinose	10.7 (0.7)	23.6 (1.3)
Rhamnose	108.8 (4.4)	45.7 (5.1)	Rhamnose	204.9 (3.7)	165.0 (3.8)	Galactose	4.5 (0.1)	5.6 (0.4)
Arabinose	96.5 (3.3)	54.3 (3.6)	Arabinose	106.0 (1.4)	103.9 (3.0)	Glucose	153.7 (3.2)	146.8 (8.9)
Galactose	141.5 (3.7)	59.4 (5.3)	Galactose	145.2 (2.6)	119.7 (3.8)	Mannose	22.2 (0.4)	21.3 (1.4)
Glucose	33.8 (1.1)	63.3 (2.3)	Glucose	45.6 (4.5)	134.8 (4.3)	Xylose	111.4 (2.2)	105.5 (6.5)
Xylose	269.3 (9.5)	115.0 (11.4)	Xylose	233.8 (4.8)	184.6 (5.6)			
GalA	127.4 (4.2)	51.5 (5.3)	GalA	98.4 (2.1)	77.9 (2.7)			
Total sugars	811.9 (26.1)	402.9 (30.5)	Total Sugars	884.1 (11.0)	817.4 (23.2)	Total sugars	304.2 (5.2)	303.7 (18.3)
Methyl ester content	0.28 (0.04)	0.60 (0.09)	Methyl ester content	2.6 (0.32)	0.85 (0.04)			
% Protein	2.15 (0.82)	21.40 (8.48)						

Monosaccharides are expressed as mg g^-1^ (of AIR, EPF or EHP per gram of seed). Methyl ester content is expressed as mmol of methanol per gram AIR or EPF, and protein content is represented as w w^-1^ percentage. Protein content was not measured in EPF and EHF.

It is well established that RG-I from FM is branched (Fedeniuk and Biliaderis, 1994; Macquet et al., 2007; Elboutachfaiti et al., 2017; Ding et al., 2018). Differences in branching between cultivars are suggested by the molar ratios of specific sugars: Ara/Rha (0.57 vs. 0.69), and Gal/Rha (0.65 vs. 0.66) in Kallfu and Wenutram, respectively. These ratios indicate a higher degree of RG-I branching in Wenutram with Arabinan side chains (**Supplementary Table 2**). Pectin methylation is a key modification influencing mucilage properties (Saez-Aguayo et al., 2013). To assess differences in HG methylesterification, methyl ester content was measured. Wenutram showed higher methyl ester levels in AIR, while Kallfu had more in the EPF (**Table 1**). Due to better representation of FM pectins, all subsequent references will focus on the EPF. This variation may significantly affect HG modification (Levesque-Tremblay et al., 2015) and overall FM structure. due to better representation of FM pectins, all subsequent references will focus on the EPF. due to better representation of FM pectins, all subsequent references will focus on the EP, glucose and xylose were predominant, comprising 86.6% in Kallfu and 83% in Wenutram, followed by mannose (7.3% and 7%, respectively). These values were similar across cultivars. Importantly, mannose was detectable only after hemicellulose enrichment. Wenutram showed higher arabinose and galactose levels than Kallfu (**Table 1**).

### Detection of polysaccharides domains in FM showed contrasting structures between Kallfu and Wenutram

**Figure 2:**
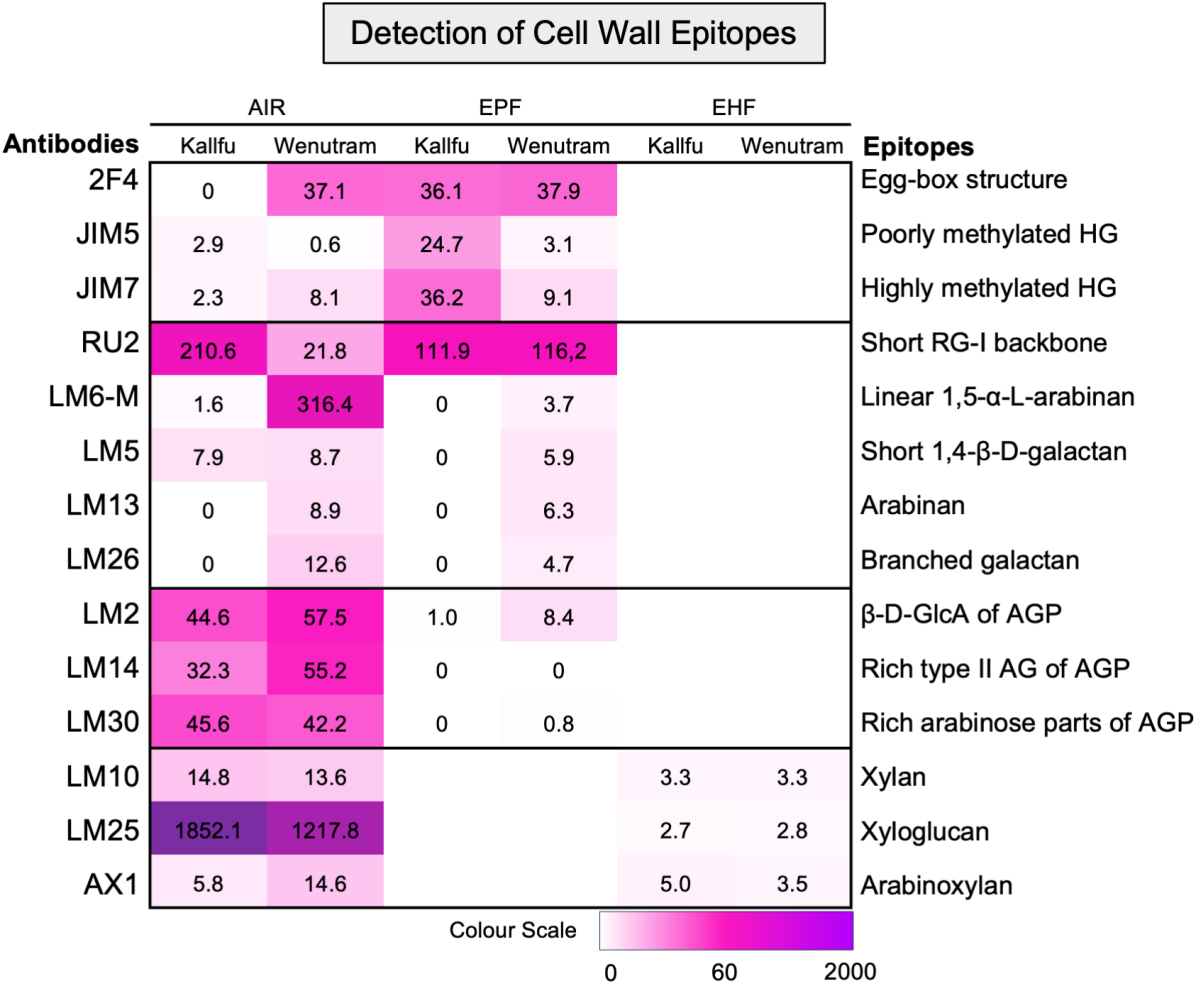
Detection of specific cell wall epitopes in AIR, EPF, and EHP from Kallfu and Wenutram mucilage. Heatmap generated from dot blot assay using a panel of antibodies to detect cell wall epitopes in mucilage AIR, EPF and EHP. Dot intensities were quantified using ImageJ, and the weighted values were represented in a heatmap (for details, see **Supplementary Figure 1**).

To identify the different polysaccharide domains present in FM, the AIR, EPF, and EHF were analyzed using antibodies specific to specific epitopes. First, HG was analyzed for its methylation pattern and the presence of egg-box structure. In AIR, this structure was detected only in Wenutram, whereas in the EPF, the epitope was detected at similar intensities in both cultivars (**Figure 2**). Poorly methylated HG showed higher detection in Kallfu compared to Wenutram in both AIR and EPFs. Conversely, highly methylated HG showed greater detection in Wenutram’s AIR but was more strongly detected in Kallfu’s EPF (**Figure 2**). These results suggest that the HG in Kallfu’s EPF is more highly methylated, consistent with earlier findings of higher methyl ester content in this fraction (**Table 1**).

RG-I structure was assessed through detection of its unbranched backbone and specific side branches. Unbranched RG-I was more abundant in Kallfu’s AIR, though no differences were found in the EPF (**Figure 2**). Side-branch analysis using LM6-M and LM13 (Cornuault et al., 2017; Pedersen et al., 2012) revealed higher levels of α-1,5-arabinan in Wenutram. Similarly, LM5 and LM26 antibodies (Pedersen et al., 2012; Torode et al., 2018) indicated stronger signals for linear β-1,4-galactan and β-1,6-galactosyl substitutions in Wenutram. Together, these results suggest that RG-I is more branched in Wenutram than in Kallfu (**Figure 2**).

Arabinogalactan-proteins (AGPs) were analyzed using three antibodies: LM2 (β-d-GlcA terminal in galactans; Smallwood et al., 1996), LM14 (type II AG-rich epitopes; Moller et al., 2008), and LM30 (AGP-rich arabinose regions; Wilkinson et al., 2017). Strong detection of all three was observed in the AIR of both cultivars, while labeling was nearly absent in Kallfu’s EPF (**Figure 2**).

Hemicellulose domains were assessed in AIR and EHFs. Xylan, detected with LM10 (McCartney et al., 2005), showed weak but consistent labeling in both cultivars. Xyloglucan, detected with LM25 (Pedersen et al., 2012), had stronger labeling in AIR, especially in Kallfu. Arabinoxylan was identified with AX1 (Guillon et al., 2004) in both AIR and EHFs, with slightly higher intensity in Wenutram AIR. These findings confirm the presence of at least three hemicellulose domains in FM (**Figure 2**).

**Figure 3:**
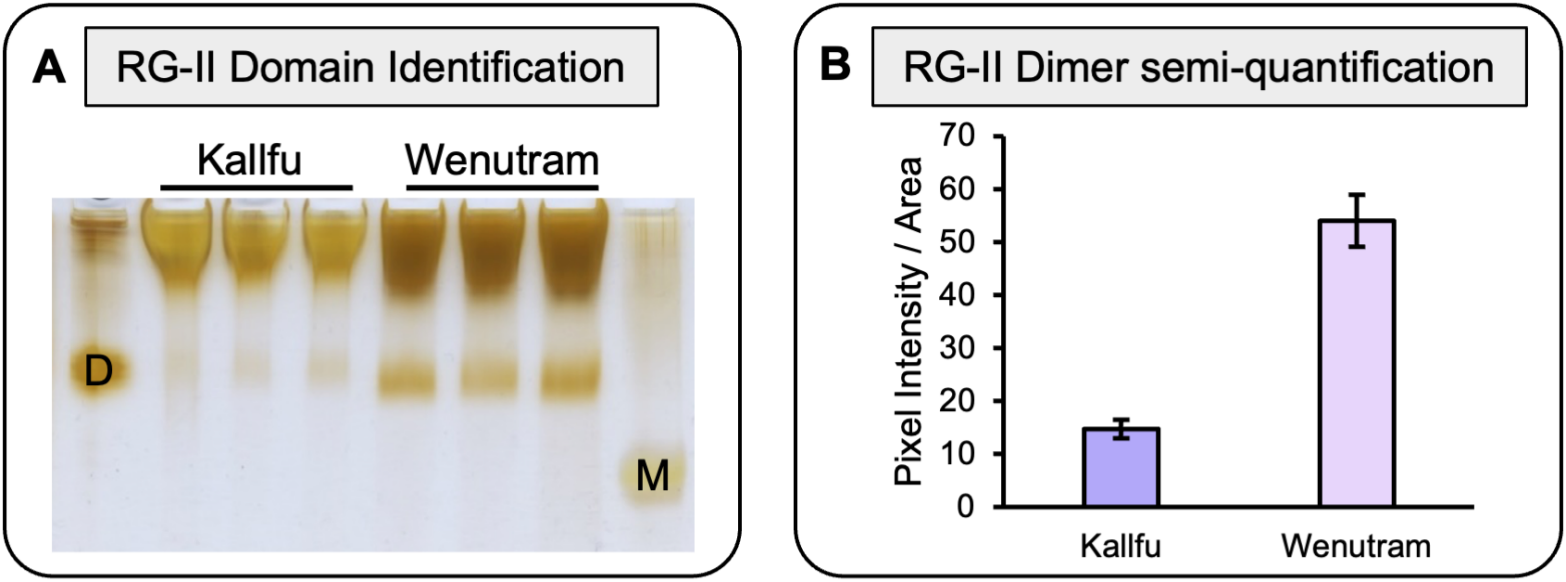
Analysis of RG-II isolated domains from Kallfu and Wenutram mucilage. **(A)** Detection of the RG-II domain by electrophoresis. 2 mg AIR was saponified and digested with endo-PG in 100 μL of digestion buffer, and then 8 μL the supernatant was analyzed using polyacrylamide electrophoresis. Standards (10.8 μg each) were used to identify the RG-II domain in its dimeric D and monomeric M forms. **(B)** RG-II Dimer relative quantification. Pixel intensity and Area of each band was quantify using ImageJ.

RG-II presence was further analyzed using PAGE, revealing the presence of RG-II dimers in both cultivars. Wenutram exhibited a higher abundance of dimeric RG-II, while Kallfu showed negligible amounts (**Figure 3**). This result suggests that the greater abundance of dimeric RG-II in Wenutram may contribute to a stiffer mucilage structure compared to Kallfu.

### Wenutram mucilage has a more branched and complex RG-I than Kallfu

**Figure 4:**
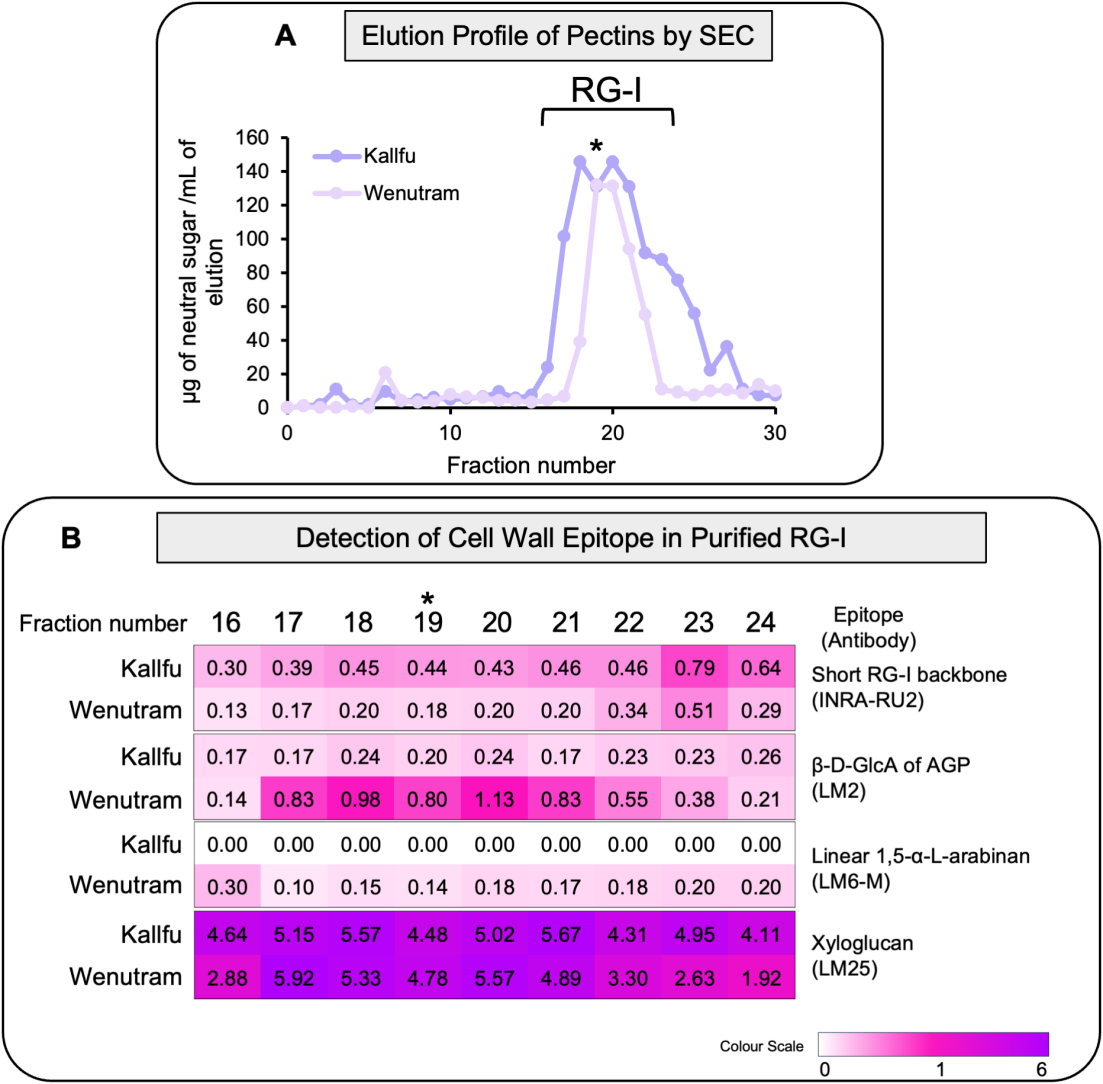
Analysis of RG-I isolated domains from Kallfu and Wenutram seed mucilage. **(A)** Elution profile of the EPF RG-I domain isolated from size exclusion chromatography (SEC). The EPF was saponified, digested with endoPG, and the different domains were separated by size exclusion chromatography. The profile was generated by quantifying neutral sugars in each collected fraction, from 0 to fraction 30. The graph corresponds to one sample of EPF of Kallfu and Wenutram as an example. The analyzed fractions were selected based on each elution profile, with the fraction containing the highest neutral sugars content (indicated by an asterisk) chosen as the central point for analysis. Three fractions preceding and five fractions following the peak were included. **(B)** Dot Blot Analysis of Specific RG-I Epitopes in Isolated RG-I Fractions. The RG-I backbone was detected using the INRA-RU2 antibody; arabinogalactan proteins were identified with LM2; arabinan with LM6-M; and xyloglucans with the LM25 antibody.

To further investigate differences in RG-I branching between cultivars, EPF from Kallfu and Wenutram were subjected to SEC to separate RG-I from HG and RG-II. RG-I fractions were collected (**Figure 4A**), and different cell wall (CW) epitopes were analyzed. Detection of the RG-I revealed a higher abundance of unbranched RG-I in Kallfu compared to Wenutram (**Figure 4B**).

The trend observed in arabinan detection from AIR and EPFs was consistent with the RG-I-enriched eluates: Wenutram contained more 1,5-α-arabinan than Kallfu. This finding correlated with the lower detection of unbranched RG-I, suggesting that Wenutram has a more branched RG-I structure than Kallfu.

Additionally, higher levels of AGPs were detected in Wenutram’s RG-I-enriched eluates compared to Kallfu (LM2), indicating a more complex branched RG-I structure (**Figure 4B**). Unexpectedly, a substantial proportion of xyloglucan was also detected in the RG-I-enriched eluates of both cultivars. This finding aligned with the varying amounts of xyloglucan detected between AIR and the EHF (**Figure 4B**).

### Pectin domains exhibit distinct distributions in the flaxseed MSCs

**Figure 5:**
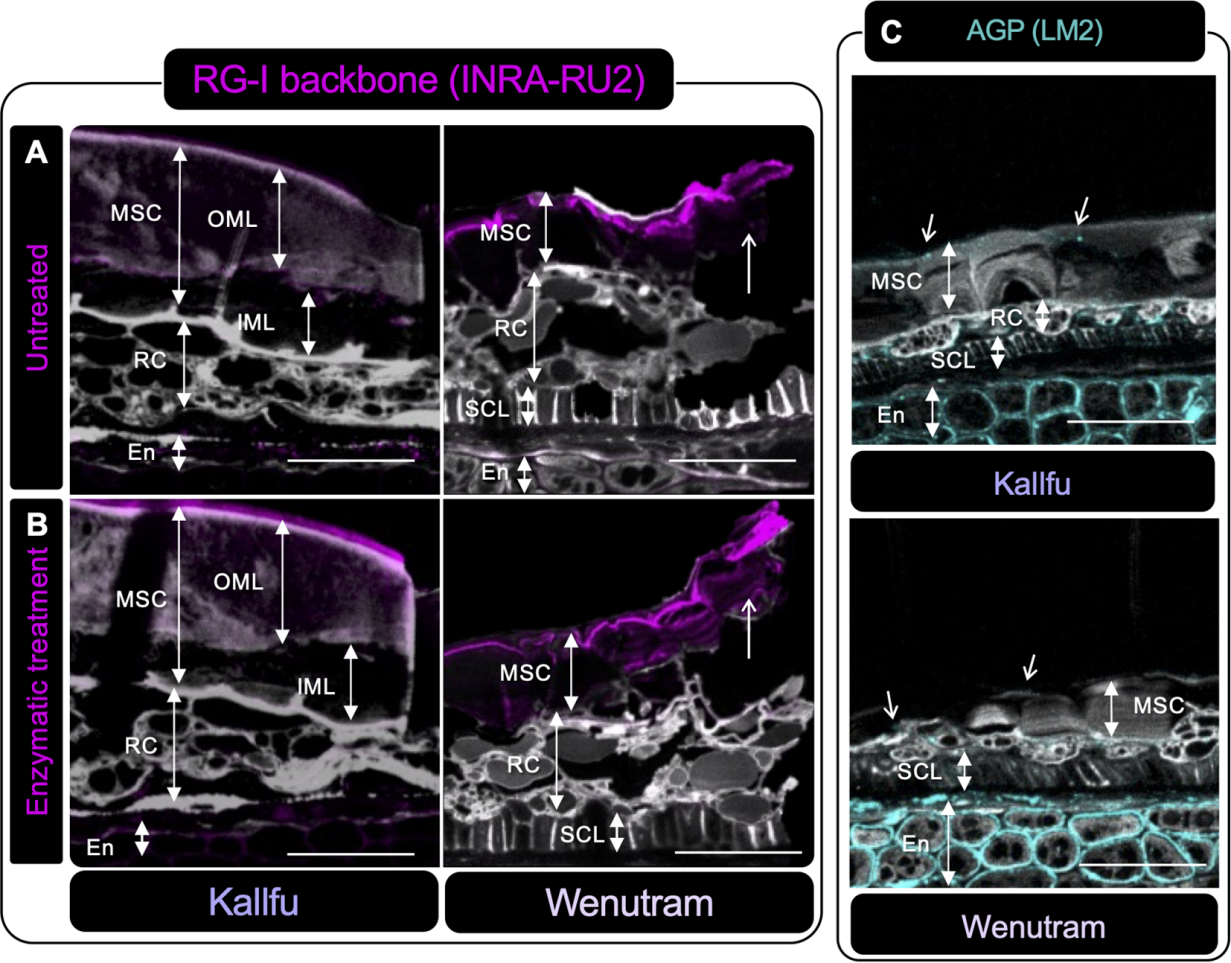
Immunolabeling of RG-I and AGP in seed sections of Kallfu and Wenutram seed coat. **(A, B)** Immunolabeling of the RG-I backbone using the INRA-RU2 antibody. Confocal microscopy optical section reconstruction of flax seed coat. In panel **(A)** an intact RG-I structure is observed, whereas **(B)** shows a section digested with arabinofuranosidase, an enzyme that removes RG-I side chains. The enzymatic treatment increased both the intensity and the area of labeling in both varieties, indicating that arabinan chains are linked to RG-I, as more epitopes were exposed following digestion. Arrows highlight differences in RG-I detection between undigested and digested sections. **(C)** The presence of arabinogalactan proteins (AGPs) was detected using the LM2 antibody. Labeling for AGPs was predominantly localized in the endosperm, with minimal labeling in other structures. Propidium iodide (PI) (in gray) was used for general visualization. Identified structures include mucilage secretory cells (MSC), the outer mucilage layer (OML), the inner mucilage layer (IML), ring cells (RC), the sclerotic cell layer (SCL), and the endosperm (En). The scale bar represents 25 μm.

Immunolabeling on resin-fixed seed sections was used to localize polysaccharide domains in the seed coats of Kallfu and Wenutram. Unbranched RG-I was detected in both cultivars (**Figure 5A**). In Kallfu, it was homogeneously distributed across the MSC, particularly in the outer mucilage layer (OML), while in Wenutram it was mainly localized to the outermost MSC region. Digestion with arabinofuranosidase, which prunes arabinan chains, enhanced RG-I backbone detection in both cultivars—most notably in Wenutram’s outer mucilage (**Figure 5B**). Although the enzyme appears to expose RG-I, whether this results from partial trimming or full removal of side branches remains unclear. Combined with dot blot results, this supports that RG-I is more branched in Wenutram than in Kallfu.

AGP distribution, assessed with LM2, was mainly detected in the endosperm apoplast in both cultivars, with slightly higher intensity in Wenutram. A dotted pattern was observed in Kallfu’s MSC, sclerotic cell layer (SCL), and ring cells (RC) (**Figure 5C**).

**Figure 6:**
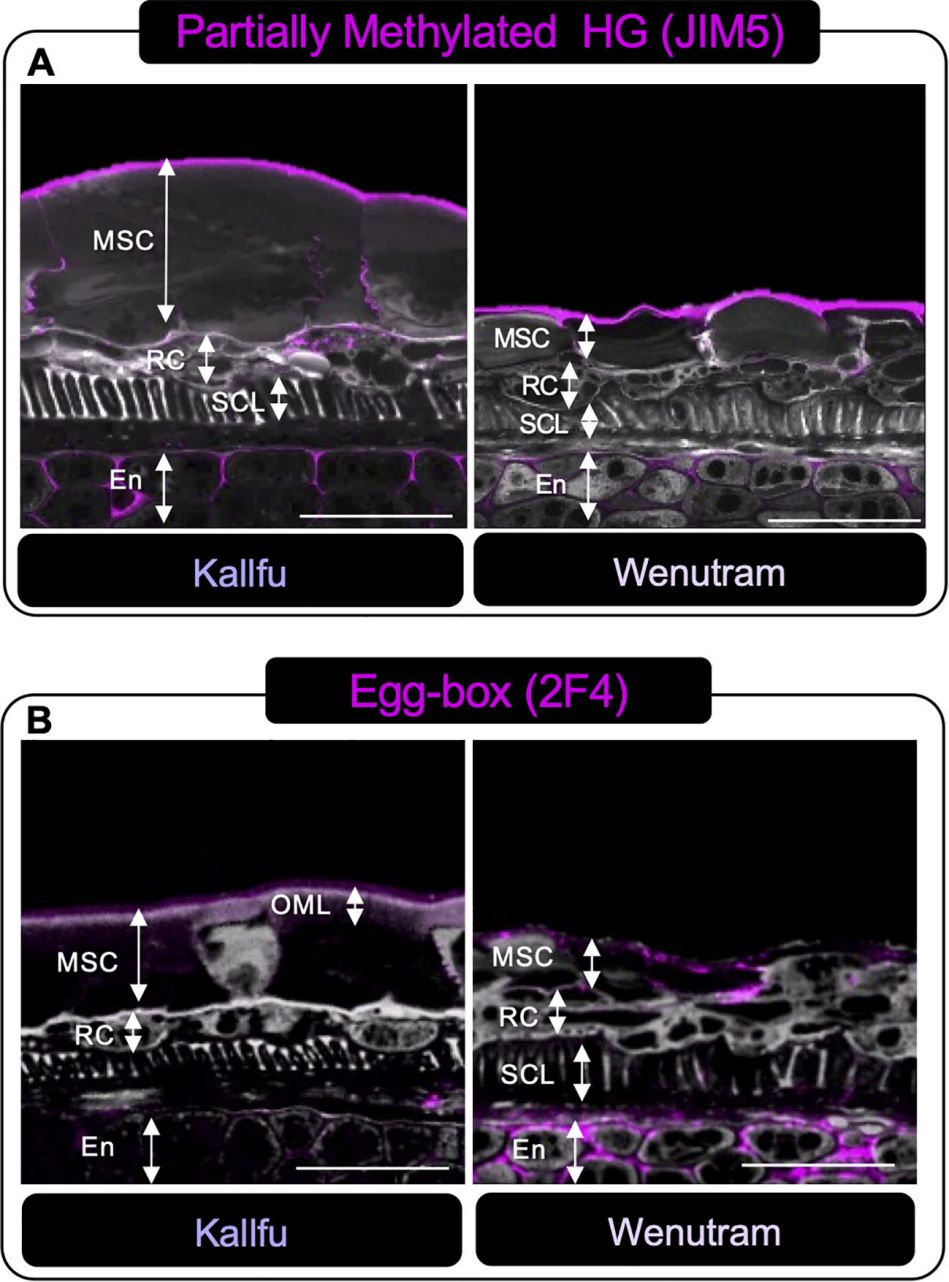
Immunolabeling analysis of homogalacturonan distribution in Kallfu and Wenutram Seed Coats sections. Confocal microscopy optical section reconstruction of flax seed coat. **(A)** Partially methylated HG pattern detection using JIM5 antibody. The labeling distribution was similar between the two varieties, predominantly located in the endosperm and along the outer border of the mucilage secretory cells. **(B)** Distribution of Egg-Box structures determined using the 2F4 Antibody. Labeling was particularly intense in the endosperm of Wenutram. Propidium iodide (PI) (in gray) was used for general visualization. Key structures, including mucilage secretory cells (MSC), the outer mucilage layer (OML), ring cells (RC), the sclerotic cell layer (SCL), and the endosperm (En), were identified. The scale bar represents 25 μm.

The label of poorly methylated HG was primarily detected in the outermost part of the MSC, as well as at the interface between two MSCs and in what appears to be the apoplast of the endosperm (**Figure 6A**). The presence of the egg-box structure was detected in the OML of Kallfu and in the outermost part of the MSC and the apoplast of the endosperm in Wenutram (**Figure 6B**).

**Figure 7:**
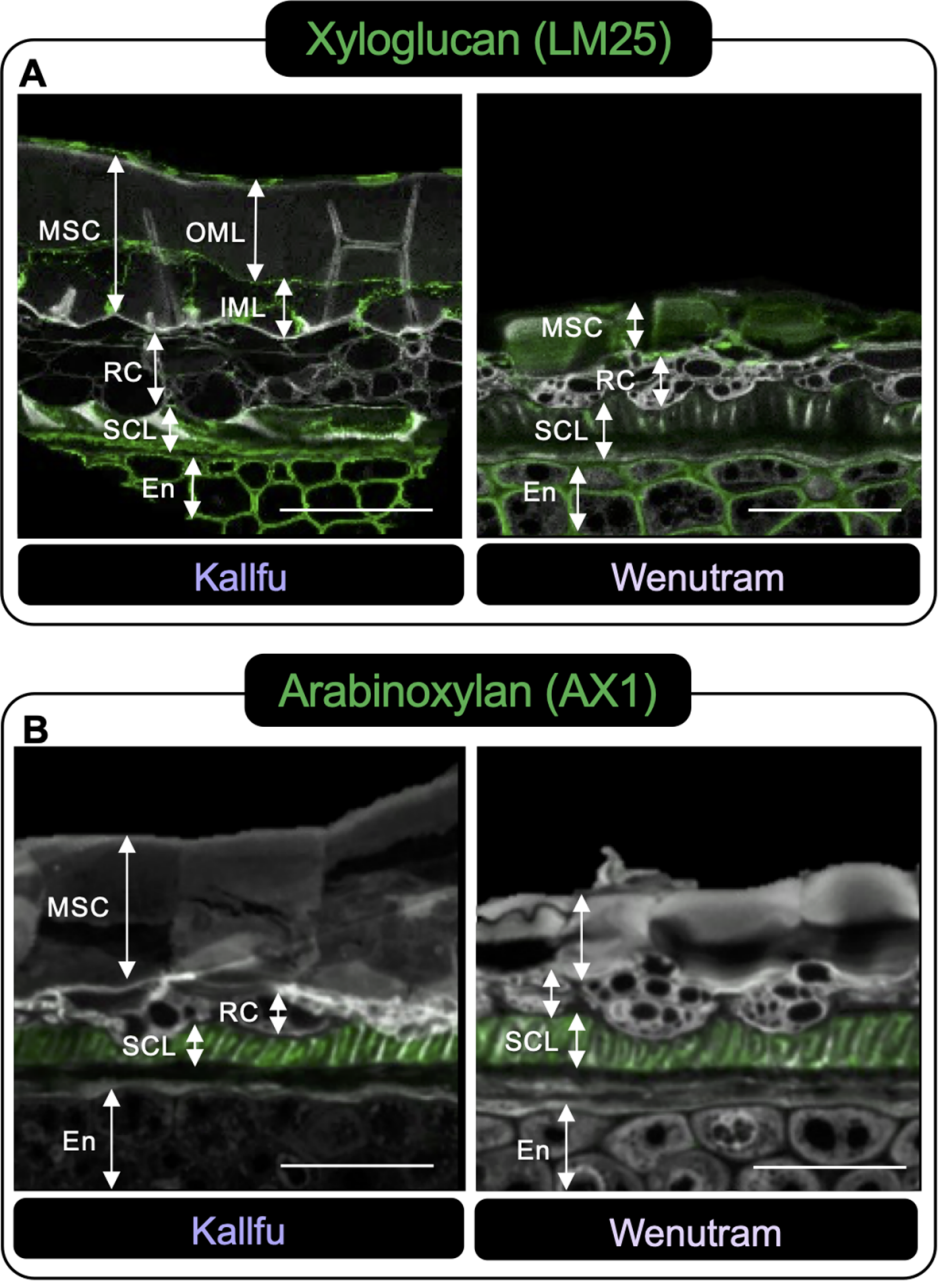
Distributions of Hemicellulosic domains in seed sections of Kallfu and Wenutram seed coat. Confocal microscopy optical section reconstruction of flax seed coat. **(A)** Xyloglucan analysis using LM25. The labeling of xyloglucans in the endosperm was similarly distributed in both varieties. However, in the mucilage secretory cells, the signal differed: it was restricted to specific areas in Kallfu but appeared more broadly spread in Wenutram. **(B)** Arabinoxylan detection using AX1. Labeling with this antibody showed a comparable distribution and intensity in both varieties. Propidium iodide (PI) (in gray) was used for general visualization. Key structures, including mucilage secretory cells (MSC), outer mucilage layer (OML), inner mucilage layer (IML), ring cells (RC), the sclerotic cell layer (SCL), and endosperm (En), were identified. The scale bar represents 25 μm.

Xyloglucan (LM25) and arabinoxylan (AX1) were also examined. Xyloglucan showed similar patterns in both cultivars, with signal in the MSC and endosperm apoplast (**Figure 7A**). In Kallfu, it was detected at the boundaries of the outer mucilage layer (OML) and the inner mucilage layer (IML), particularly at the separation between the cells. In contrast, Wenutram displayed a homogenous distribution of xyloglucan across all MSCs. Arabinoxylan labeling was restricted to the SCL in both cultivars, with no signal in MSCs (**Figure 7B**), suggesting epitope masking due to steric hindrance.

### Wenutram mucilage accumulates more CW proteins compared to Kallfu mucilage

The total protein content in the AIR was measured for both cultivars (**Table 1**). Wenutram exhibited a higher protein content, with approximately 20% of its AIR consisting of proteins, while only 2.5% of Kallfu’s AIR was protein.

To further investigate the composition of FM, we performed a proteomic analysis of Kallfu and Wenutram mucilage. Protein analysis of FM from both cultivars identified 527 protein groups. The minimum criteria for protein identification were the presence of at least two unique and exclusive peptides. Since not all proteins were consistently found in every technical replicate, only those present in all the replicates for each cultivar were considered. From this analysis, 256 protein groups were identified: 19 unique to Kallfu, 136 unique to Wenutram, and 101 common to both. This indicates that not only the total number of proteins differs between cultivars, but also the nature of these proteins (**Supplementary Figure 2A**). To gain insights into the functions of the common proteins, we performed a Gene Ontology (GO) term enrichment analysis (**Supplementary Figure 2B**). The main functions identified were catalytic activity and binding. Further analysis using GOATOOLS revealed that the molecular functions overrepresented in both cultivars were catalytic activity, hydrolase activity, and binding (**Supplementary Figure 5**). The protein groups unique to Kallfu, due to the low peptide quantification, did not meet the p-value cutoff for identifying overrepresented molecular functions. In contrast, the protein groups unique to Wenutram were primarily associated with structural molecule activity (**Supplementary Figure 6**).

Next, we focused on the differentially quantified proteins between the two cultivars, identifying 85 protein groups with differential abundance (**Supplementary Figure 7**). The overrepresented functions in these 85 groups were structural molecular activity, structural constituent of ribosome, and enzyme inhibitor activity (**Supplementary Figure 8**).

**Figure 8:**
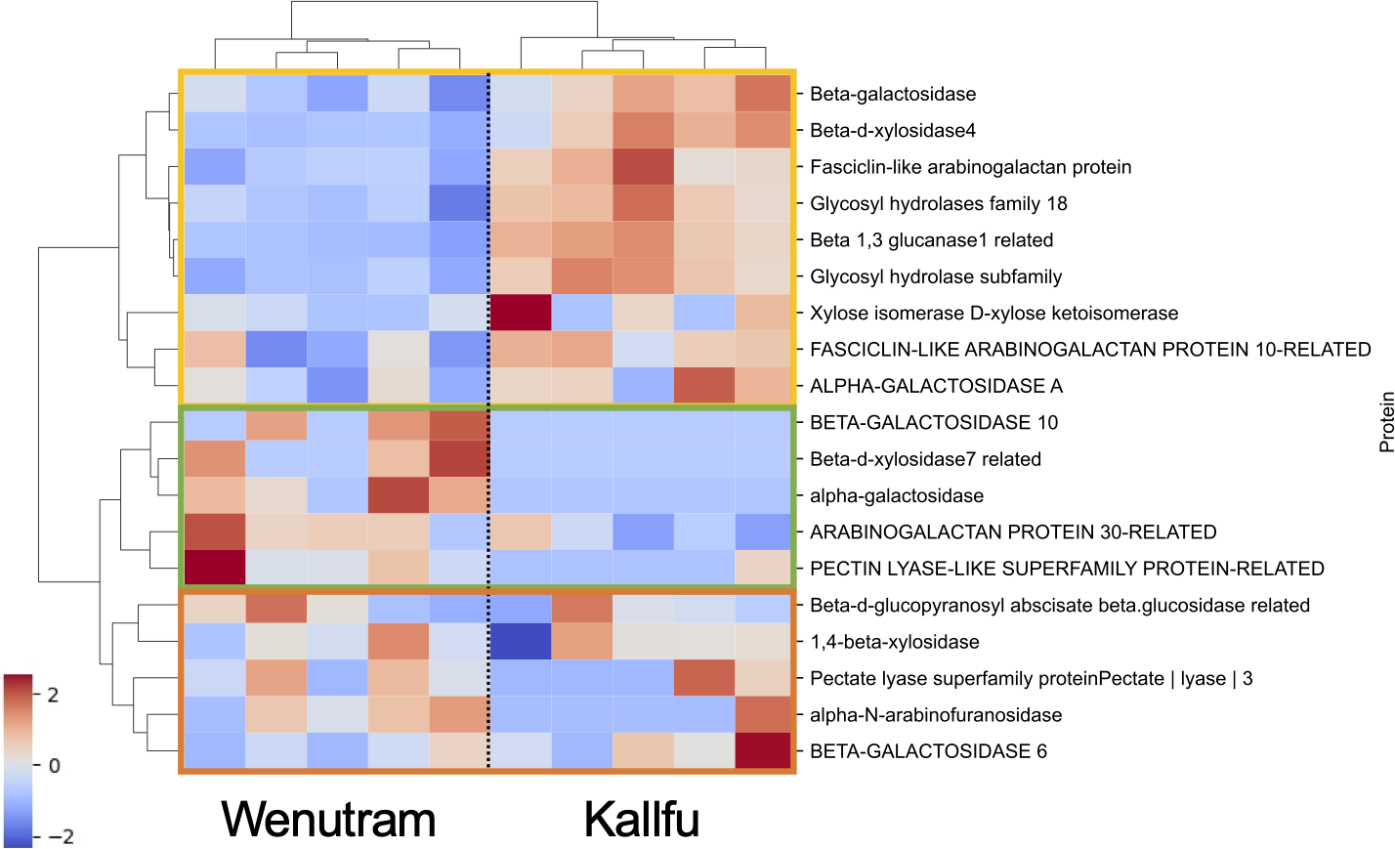
Heatmap of proteins associated with CW sugar metabolism. The heatmap illustrates proteins involved in CW sugar metabolism. The first section (yellow box) highlights proteins that are more abundant in Kallfu, the second section (green box) indicates proteins more abundant in Wenutram, and the third section (orange box) shows proteins with similar accumulation levels in both cultivars. Values are presented on a z-score scale.

We arbitrarily selected a group of proteins associated with various enzymatic activities related to CW polysaccharides. In total, 19 proteins were identified and organized in a heatmap (**Figure 8**). Three distinct sections are distinguishable: the first section (yellow box) includes proteins that are more abundant in Kallfu, mainly associated with hydrolase activity, except for the FASCICLIN-LIKE ARABINOGALACTAN PROTEIN 10-LIKE that belong to the FASCICLIN-LIKE ARABINOGALACTAN PROTEIN (FLA) family which are classified as a subclass of chimeric AGPs (He et al., 2019). The second section (green box) contains proteins predominantly quantified in Wenutram, including four proteins with hydrolase activity and one AGP (ARABINOGALACTANPROTEIN 30-RELATED). The last section (orange box) consists of proteins with similar abundance in both cultivars, which indicates that there are some common functions besides the structural difference. Three of these proteins have hydrolase activity (1,4-BETA-XYLOSIDASE, ALPHA-N-ARABINOFURANOSIDASE AND BETA-GALACTOSIDASE 6), there is also a PECTATE LYASE, all of these are related to CW modification. However, BETA-D-GLUCOPYRANOSYL ABSCIATE BETA-GLUCOSIDASE RELATED is associated with abscisic acid activation (Ma et al., 2018).

These results suggest a differential accumulation of CW proteins in the analyzed flax cultivars. Notably, Kallfu shows a greater accumulation of proteins related to hydrolase activity than Wenutram, particularly associated with polysaccharides modification like GALACTOSIDASE, XYLOSIDASE, GLYCOSY HYDROLASE, and GLUCANASE, while simultaneously exhibiting a less branched RG-I structure (**Figures 3**, **5**).

Finally, the top 20 most quantified proteins for each cultivar were analyzed. Among these, 11 proteins were present in both cultivars, while 9 were exclusive to either Wenutram or Kallfu. Of the proteins common to both cultivars, three have catalytic functions related to polysaccharide modification: BETA-1,3-GLUCANASE-1-RELATED, GLYCOSYL HYDROLASE, and ALPHA-MANNOSIDASE. The presence of these proteins highlights the significance of polysaccharide modification in the mucilage of both Kallfu and Wenutram (**Table 2**). In Kallfu, additional proteins associated with CW modification were identified, including those involved in the post-translational modification of PMEs (PECTIN METHYLESTERASES), such as PROPROTEIN CONVERTASE SUBTILISIN and SERINE PROTEASE INHIBITOR (SERPIN) FAMILY PROTEIN-RELATED (Rautengarten et al., 2008; Sénéchal et al., 2014). Additionally, a BETA-D-XYLOSIDASE 3-RELATED protein was exclusively detected in Kallfu. Conversely, Wenutram exhibited only one protein related to CW modification: FOUR ADJACENT PUTATIVE SUBTILASE FAMILY-RELATED, which may also modify PMEs. These results support the idea that Kallfu mucilage undergoes more modifications than Wenutram.

**Table 2:** Most represented protein groups in the mucilage of Kallfu and Wenutram cultivars.

ID	Name	Quantification average	
		Kallfu	Wenutram
Lus10019801	BETA-1,3-GLUCANASE 1-RELATED	28	22.8
Lus10002319	*HEAT SHOCK 70 KDA PROTEIN 5*	23.4	49.2
Lus10022070	*Cupin*	22.6	25.6
Lus10031235	GLYCOSYL HYDROLASE	22	20.2
Lus10006420	CUPIN DOMAIN-CONTAINING PROTEIN	17	21.4
Lus10009014	STRICTOSIDINE SYNTHASE-RELATED	16.2	17.2
Lus10031454	Arylformamidase/Kynurenine formamidase	14.6	17.6
Lus10023870	Fructose-bisphosphate aldolase/Fructose-1,6-bisphosphate triosephosphate-lyase	13.8	21
Lus10012871	BETA-D-GLUCOPYRANOSYL ABSCISATE BETA-GLUCOSIDASE-RELATED	13.2	27.2
Lus10029681	PROTEIN DISULFIDE ISOMERASE	12.4	25.6
Lus10041516	ALPHA-MANNOSIDASE	11	16.2
Lus10038437	Tetrahydroberberine oxidase/THB oxidase	25.2	–
Lus10009867	PROPROTEIN CONVERTASE SUBTILISIN	24.2	–
Lus10003579	ZINC FINGER FYVE DOMAIN CONTAINING PROTEIN	18.2	–
Lus10003231	CHITINASE-RELATED	17.4	–
Lus10035618	CHITINASE-RELATED	12.8	–
Lus10006302	Thaumatin family	11.8	–
Lus10032754	SERINE PROTEASE INHIBITOR (SERPIN) FAMILY PROTEIN-RELATED	10.8	–
Lus10041224	ASPARTYL PROTEASE FAMILY PROTEIN	10.8	–
Lus10020966	BETA-D-XYLOSIDASE 3-RELATED	10.2	–
Lus10039235	OF FOUR ADJACENT PUTATIVE SUBTILASE FAMILY-RELATED	–	26.2
Lus10011770	CHAPERONIN	–	24.4
Lus10015116	Aconitate hydratase/Citrate(isocitrate) hydro-lyase	–	22
Lus10004471	GLUCOSE AND RIBITOL DEHYDROGENASE HOMOLOG 1-RELATED	–	20.2
Lus10002844	Phosphopyruvate hydratase/Enolase	–	19.6
Lus10031743	PHOSPHOGLYCERATE KINASE	–	19.4
Lus10020788	UTP-glucose-1-phosphate uridylyltransferase/UDP-glucose pyrophosphorylase	–	18.2
Lus10027517	HEAT SHOCK PROTEIN 90-1	–	17.6
Lus10002620	Tetrahydropteroylglutamate-homocysteine transmethylase	–	15.4

The higher protein quantification for each cultivar is underline.

## Discussion

Kallfu and Wenutran are two flax cultivars developed in Chile that display distinct seed mucilage phenotypes. Kallfu produces roughly twice the amount of mucilage sugars compared to Wenutran; however, this difference does not directly explain the reduced mucilage extrusion observed in Wenutran (**Figure 1**). This raises the question of whether structural differences in the mucilage of these cultivars could influence the extrusion pattern observed in Wenutran.

The FM of both cultivars is mainly composed of xylose, in similar proportions to those measured in AIR and EPF, suggesting a potential role for a xylose-enriched polymers in the differences observed in mucilage extrusion. The immunolabeling analysis revealed changes in the detection of xylan, arabinoxylan, and xyloglucan; however, these differences were not substantial. Therefore, they do not explain the difference in the mucilage extrusion phenotype. Additionally, more in-depth techniques, such as linkage analyses, would be necessary to elucidate the role of xylose in FM structure (**Table 1**, **Figures 2**, **4**, **7**). Given the techniques available, one possible explanation is that the antibodies used may fail to recognize the specific polysaccharide structures present in these new cultivars. AX1 antibodies detect xylan chains with a terminal arabinose (Guillon et al., 2004); however, based on FM linkage analysis (Ding et al., 2018), the proposed structure of arabinoxylan in FM consists of a xylan chain substituted by a chain of three residues, with the primary terminal residue being xylose. These structural differences could result in reduced detection of these polysaccharides.

Although RG-I is the primary constituent of FM, it has been reported that higher levels of arabinoxylan were also expected in FM (Warrand et al., 2005; Naran et al., 2008; Elboutachfaiti et al., 2017; Ding et al., 2018), and the higher xylose content in our cultivars also suggested this. However, it was not possible to detect high levels of arabinoxylans with the tools currently at our disposal.

When EPFs of both cultivars are compared, Wenutram contains less rhamnose and galacturonic acid than Kallfu; however, arabinose levels are similar in both cultivars (**Table 1**). This finding, combined with the higher detection of arabinans in Wenutram compared to Kallfu (**Figures 2**, **4**) and the increased detection of RG-I in samples treated with arabinofuranosidase (**Figures 5A, B**), strongly confirm that RG-I from Wenutram mucilage contains more arabinan chains.

Alterations in RG-I structure, more particularly related with changes on side branches, composed of arabinose and/or galactose, are associated with difficulty in releasing mucilage in Arabidopsis (Macquet et al., 2007). The *BXL1* gene encodes a bifunctional β-d-xylosidase/α-l-arabinofuranosidase that cleaves the arabinan side chains from RG-I (Arsovski et al., 2009; Williams et al., 2020). The impact of its absence is evident in the *Atbxl1* mutants, which show slow and patchy mucilage release (Arsovski et al., 2009), related to hairy RG-I contained in the mucilage pocket, similar to what is observed in Wenutram seeds (Williams et al., 2020). Similarly, when *MUM2 (MUCILAGE MODIFIED 2)*, which encodes a β-galactosidase, is not functional, Arabidopsis seeds cannot extrude mucilage, even though mucilage is correctly produced during seed development (Dean et al., 2017; Macquet et al., 2007). Although the exact structure of FM´s RG-I side branches remains unclear, it has been proposed that they may consist of two different polysaccharides: one composed of l-fucose, d-xylose, and d-galactose, and the other consisting of the same monosaccharides plus d-arabinose (Warrand et al., 2005). The latter hypothesis is supported by digestion with arabinofuranosidase, which hydrolyzes α-1,2- and α-1,3-linked l-arabinofuranose residues from arabinoxylans and arabinans in the Kallfu and Wenutram MSC. This digestion results in higher detection of unbranched RG-I, primarily in Wenutram (**Figure 5A**). These results, along with the Ara/Rha molar ratio (**Supplementary Table 1**) suggest not only the presence of arabinose in the RG-I side branches, but also that arabinose side branches are more abundant in Wenutram MSC than in Kallfu. Additionally, the analysis of the RG-I-enriched fractions revealed higher levels of arabinan, AGPs, xyloglucan, and arabinoxylan in Wenutram than in Kallfu, supporting the idea that Wenutram has a more branched RG-I structure than Kallfu. However, a more detailed analysis of the RG-I structure is required to determine the exact structure of these ramifications.

FM is primarily known for its high content of RG-I, but it also contains smaller amounts of HG. Both Kallfu and Wenutram follow this composition pattern, as indicated by the high molar ratios of rhamnose to galacturonic acid in the EPF, 2.46 and 2.51, respectively (**Supplementary Table 1**). These results suggest a low abundance of HG and a potential presence of homorhamnan in the EPF, which should be confirmed through isolation of this fraction or by performing linkage analysis as reported in Qian et al., 2012. Nevertheless, despite the low abundance, HG domains were detected using the JIM5, JIM7, and 2F4 antibodies. The identification of these domains concentrated in the distal wall of MSC highlights the significance of these minor constituents in FM as observed for Chilean papaya mucilage (Sanhueza et al., 2024). This region of the MSC plays a pivotal role in mucilage release when seeds are immersed in water. *In vitro* analyses have shown that HG with lower methylation levels is more prone to forming the egg-box structure, thereby contributing to the gel’s strength (Chan et al., 2017). Additionally, the reduced methyl ester content in the EPF of Wenutram mucilage may facilitate the formation of more egg-boxes (**Table 1**). Therefore, it is plausible to propose that Wenutram has a sturdier outer layer in the MSC, potentially accounting for the patchy mucilage extrusion halo (**Figure 1B**). However, it is important to underscore that the presence of EDTA do not release mucilage completely (**Figure 1**) indicating that others CW domains are also playing a role in mucilage extrusion. While both cultivars exhibit similarities in their EPFs, differing levels of HG methylation suggest distinct variations in mucilage strength and extrusion dynamics.

RG-II is a complex domain composed of 13 monosaccharides, which can give rise to six different side branches. Side branch A can bridge two RG-II molecules through a boron ion, forming dimeric RG-II (dRG-II), which significantly strengthens the CW (Delmer et al., 2024). Remarkably, this pectin domain has been identified in FM for the first time (**Figure 3**). Furthermore, the detection of dRG-II was found to be higher in Wenutram compared to Kallfu (**Figure 3**). The study of the seed coat mucilage in the *mur1* mutant provides insights into the presence of dRG-II and its role in maintaining mucilage structure (Shi et al., 2017). The observed phenotype in the *mur1* mutant is attributed to a reduced amount of dRG-II. After vigorous agitation, the mucilage in the *mur1* mutant nearly vanished, leaving behind a very thin mucilage layer. This phenomenon was not observed in WT seeds, highlighting the importance of dRG-II in maintain mucilage structure and its influence in mucilage release. This observation aligns with the mucilage release patterns seen between the two cultivars: Kallfu, which releases more mucilage, exhibits negligible levels of dRG-II, while Wenutram demonstrates higher levels of dRG-II (**Figures 1A**, **3C**). Despite the alterations in HG and RG-II, a direct link between these changes and mucilage halo extrusion remains elusive. Nonetheless, these findings support the idea that Wenutram synthesizes a stiffer and compact mucilage than Kallfu.

The analysis of monosaccharide composition in the AIR and EHFs strongly suggests the presence of xyloglucan, a hemicellulose associated with CW rigidity (Hayashi and Kaida, 2011). Xyloglucan was previously overlooked by other extraction methods (Warrand et al., 2005) or primarily associated with flaxseed meal and flaxseed kernel (Warrand et al., 2005; Ding et al., 2018). These findings are consistent with the dot blot analysis, particularly for AIR, where the signal intensity was notably stronger compared to other antibodies tested for hemicellulose. Additionally, SEC analysis of EPFs reveals a close association between RG-I and xyloglucan, as RG-I co-eluted with xyloglucan. It has been reported that approximately 30-70% of xyloglucan is covalently bound to RG-I, with its synthesis occurring using RG-I as a primer for the polymerization (Popper and Fry, 2005, 2008). Immunolabeling further demonstrates that xyloglucan is localized in structural boundary regions, such as the borders of the MSC, as well as in the OML and IML of Kallfu. In contrast, in Wenutram, xyloglucan is more ubiquitously distributed within the MSC (**Figure 7A**). This distribution suggests that xyloglucan plays a significant role in reinforcing the MSC of both cultivars, providing structural support at the boundaries of the layers in Kallfu and contributing to a more rigid, homogenously distributed mucilage in Wenutram. Overall, these findings highlight a close relationship between RG-I, AGP, arabinoxylan, and xyloglucan in FM.

Miart et al. (2019), described the FM as a layered structure, where the first layer is rich in RG-I, while subsequent layers contain higher amounts of arabinan and glucans. In Kallfu, only two distinct layers were observed: the OML, rich in RG-I, was demarcated by HG and xyloglucan, whereas the IML, delimited similarly to the first, lacked identifiable domains within its boundaries (**Figures 6A**, **7A**). Conversely, Wenutram exhibited a distinct MSC structure, characterized by a single layer containing RG-I and xyloglucan, bordered by HG (**Figures 6A**, **7A**). This difference in the polysaccharide domain distribution suggests a potential temporal disparity in the synthesis of RG-I and xyloglucan. In Kallfu, RG-I synthesis appears to occur earlier than in the cultivars studied by Miart et al. (2019). In contrast, Wenutram may undergo concurrent synthesis of RG-I and xyloglucan, leading to the absence of a second layer during this process. Although challenging to confirm through histological methods alone, it is plausible that differences in mucilage content between the two cultivars influence MSC structure. Studies on Arabidopsis mucilage, extensively regarded as a model for CW research (Arsovski et al., 2010; Haughn and Western, 2012), provide further insight into structural variations. Natural variants of Arabidopsis with reduced monosaccharide accumulation (Rha, GalA, Gal, and Man), also display defects in mucilage structure compared to Col-0, similar to the differences observed between Kallfu and Wenutram (Voiniciuc et al., 2015). The combined findings from cytological analyses, monosaccharide profiling, and immunolabeling underscore that Kallfu and Wenutram not only differ in mucilage content but also exhibit discrepancies in mucilage structure and polysaccharide composition.

The plant CW is a complex structure that has been primarily studied for its polysaccharides; however, CW proteins also play significant roles in its modification and assembly (Albenne et al., 2013; Jamet et al., 2006). Interestingly, the number of proteins identified in the FM of Kallfu and Wenutram (256 proteins) significantly exceeds that found in Arabidopsis (28 proteins) (Tsai et al., 2017). These differences could be associated with the significant variation in mucilage content between Arabidopsis and flax. Arabidopsis seeds contain between 20 to 25 µg of total mucilage monosaccharides per mg of seed (Voiniciuc et al., 2015), whereas flax seeds have much higher levels, with 811.9 µg mg^-1^ of seed in Kallfu and 402.9 µg mg^-1^ of seed in Wenutram. Despite this difference in protein quantity, several protein families are common to both species, including glycosyl hydrolases (e.g., β-XYLOSIDASE, β-GALACTOSIDASE), which are important for polysaccharide modifications (Dean et al., 2017; Macquet et al., 2007; Arsovski et al., 2009; Williams et al., 2020), Cupins (known as seed storage proteins) (Dunwell et al., 2004), AGPs (e.g., FASCICLIN-LIKE) a well-known CWP (He et al., 2019), and Subtilisin, which are associated with PME regulation (Rautengarten et al., 2008; Sénéchal et al., 2014).

**Figure 9:**
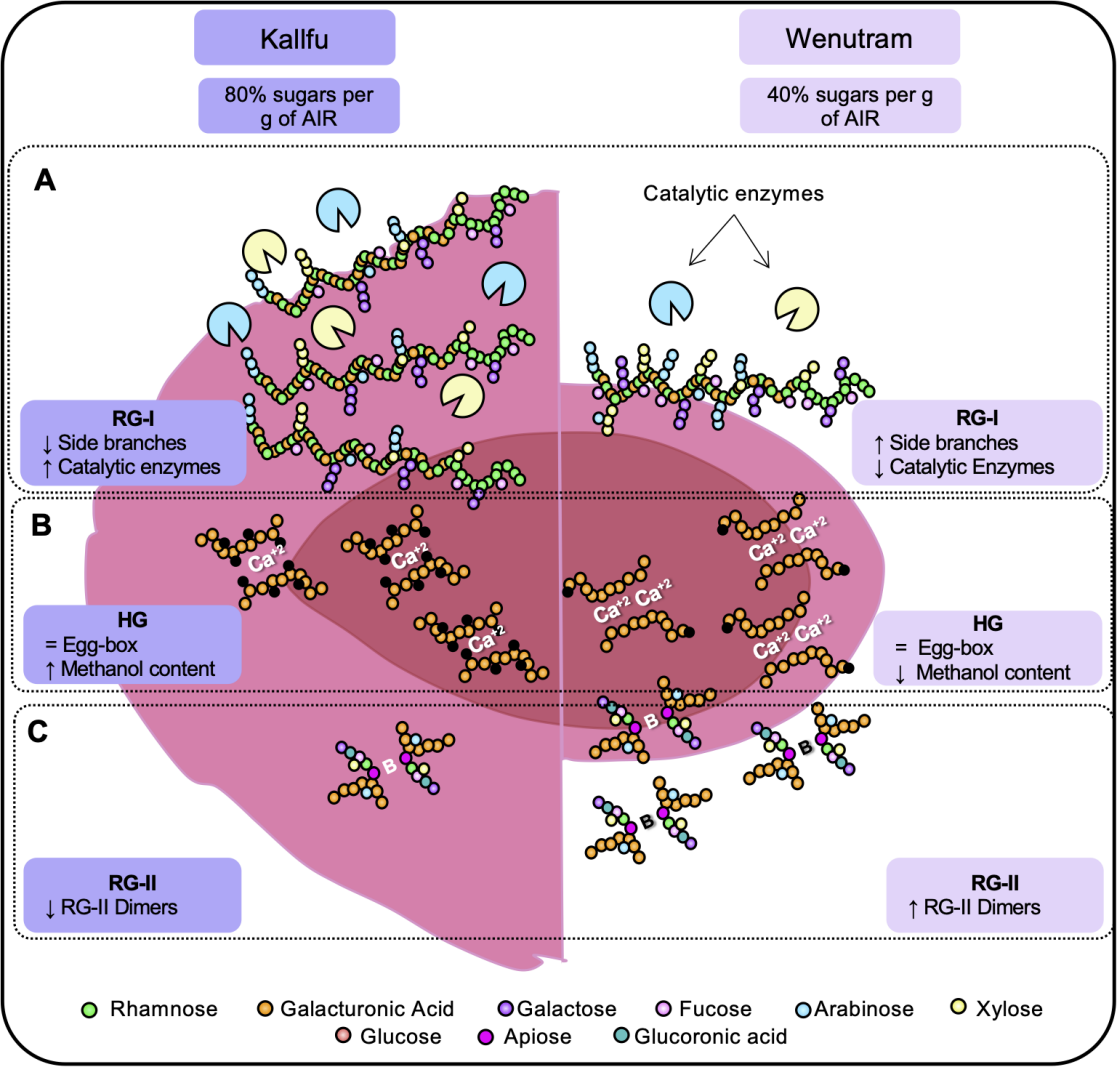
Schematic representation of the structural differences between kallfu and wenutram. This diagram summarizes the key differences identified in this study that may explain the variations in mucilage extrusion capacity between Kallfu and Wenutram. **(A)** Both cultivars are primarily composed of RG-I. Based on monosaccharide compositional analysis and immunolabeling (**Table 1**, **Figures 4**, **5**), we propose that RG-I in Wenutram contains more arabinan side chains and more AGPs than in Kallfu. **(B)** Homogalacturonan analysis revealed no significant differences in the presence of egg-box structures. However, methyl ester content is higher in Kallfu and the distribution pattern of egg-box structure varies. **(C)** Wenutram shows a higher abundance of the dimeric form of RG-II compared to Kallfu, which likely contributes to increased CW stiffening. Together, these structural differences contribute to the patchy and sparse mucilage extrusion pattern observed in Wenutram, whereas Kallfu exhibits a more fluid extrusion.

Our proteomic analysis provides important insight into this process, revealing a high accumulation of enzymes with catalytic activity in the FM of both cultivars. Among these are various hydrolases, such as GLUCANASES, GLYCOSYL HYDROLASES, GALACTOSIDASES, and XYLOSIDASES, which are likely involved in remodeling polysaccharide structures. Notably, Kallfu shows a higher abundance of these enzymes compared to Wenutram, particularly those associated with cell wall modification, including β-GALACTOSIDASE, β-D-XYLOSIDASE4, GLYCOSYL HYDROLASE, β-1,3-GLUCANASE-RELATED, and α-GALACTOSIDASE A (**Figures 8**, **9**). The elevated presence of hydrolases in Kallfu suggests active pruning of RG-I side branches, which likely reduces structural complexity and leads to a more relaxed mucilage, similar to the smooth RG-I observed in Arabidopsis (Arsovski et al., 2009; Dean et al., 2017; Macquet et al., 2007). In contrast, the lower abundance of hydrolases in Wenutram may result in reduced pruning, allowing the persistence of more side branches, thereby contributing to a more rigid and compact mucilage structure (**Figure 9**). It would be interesting to investigate potential differences in the RG-I side chains between the two varieties to gain deeper insights into the variation of cell wall-modifying enzymes, and to associate these differences with specific RG-I structural features using SEC-MALLS analysis. Additionally, future molecular studies are needed to identify the specific genes involved in RG-I pruning, with candidate genes likely including those encoding catalytic proteins more abundantly expressed in Kallfu.

The presence of proteins not directly linked to the CW is common in this type of analysis due to the destructive nature of the extraction process. These proteins, referred to as ‘non-canonical’ CWPs, can comprise up to half of the proteins identified in such experiments (Jamet et al., 2006). In this study, the seed coat was disrupted despite gentle agitation in water, resulting in embryos being observed floating. This suggests potential contamination of mucilage extractions with embryo-derived proteins. Although a filter for embryonic proteins was used during the proteomic analysis, molecular functions unrelated to CWPs were still observed (Jamet et al., 2006).

This study delved into the analysis of two Chilean flaxseed cultivars, Kallfu and Wenutram, characterized by distinct mucilage content. Our findings largely corroborate previously published data, confirming that FM is predominantly composed of branched RG-I. Despite Kallfu exhibiting nearly double the mucilage content compared to Wenutram, the extrusion halo can be up to 20 times larger in Kallfu, suggesting differences in mucilage structure between the cultivars. The discrepancies in mucilage extrusion between Kallfu and Wenutram may be attributed mostly to RG-I branching, modulated by catalytic enzymes in the FM, higher content of RG-II dimers and a more homogenous distribution of xyloglucan in the MSC of Wenutram (**Figure 9A**).

Additionally, our study identifies HG and RG-II as minor components of Kallfu and Wenutram mucilage. Despite their lower abundance, the presence of HG domains within the mucilage particularly the presence of egg-box structures in the borders of the MSC (**Figure 6**) underscores their relevance, potentially contributing to mucilage functionality (**Figure 9B**).

Moreover, the presence of dimeric RG-II (dRG-II) in FM, detected for the first time in this study, may play a crucial role in mucilage structure and release dynamics (**Figure 9C**). Furthermore, xyloglucan emerges as a key hemicellulose in both cultivars, closely associated with RG-I (possible by covalent bond) and potentially contributing to mucilage strength (Popper and Fry, 2005, 2008; Hayashi and Kaida, 2011). Lastly, differences in MSC structure between Kallfu and Wenutram highlight the complexity of FM composition and its implications for mucilage functionality. While Kallfu exhibits a stratified MSC structure with distinct layers, Wenutram displays a singular layer structure, potentially indicating temporal disparities in RG-I and xyloglucan synthesis. The differences observed between Kallfu and Wenutram FM could inform future studies on pharmaceutical bioencapsulation. Kallfu, being less rigid, may offer advantages for drug delivery, while Wenutram, being more rigid, could provide greater protection for drugs requiring enhanced stability.

In summary, our comprehensive analysis underscores the intricate composition and structure of FM, shedding light on the molecular mechanisms underlying mucilage differences between flax cultivars.

## Data Availability

The datasets presented in this study can be found in online repositories. The names of the repository/repositories and accession number(s) can be found in the article/**Supplementary Material**.
