# Supplementary material for: Kallfu and Wenutram: two Chilean flaxseed varieties with contrasting mucilage production, composition, and structure
Source: Front Plant Sci. 2025 Aug 28;16:1626044. doi: 10.3389/fpls.2025.1626044 (PMC12424233; doi:10.3389/fpls.2025.1626044)
Supplement: Supplementary file 1 [file DataSheet1.pdf]

Supplementary Table 1: GO terms associated with seed.

Class	ID	Term
BP	GO:0009790	embryo development
BP	GO:0009791	post-embryonic development
BP	GO:0009793	embryo development ending in seed dormancy
BP	GO:0048316	seed development
BP	GO:0010162	seed dormancy process
BP	GO:0009793	embryo development ending in seed dormancy
BP	GO:0019915	lipid storage
BP	GO:0006629	lipid metabolic process
BP	GO:0006631	fatty acid metabolic process
BP	GO:0006633	fatty acid biosynthetic process
BP	GO:0006635	fatty acid beta-oxidation
BP	GO:0055088	lipid homeostasis
BP	GO:0046890	regulation of lipid biosynthetic process
BP	GO:0044255	cellular lipid metabolic process
BP	GO:0044242	cellular lipid catabolic process
BP	GO:0034440	lipid oxidation
BP	GO:0016042	lipid catabolic process
BP	GO:0008610	lipid biosynthetic process
BP	GO:0006869	lipid transport
BP	GO:0006629	lipid metabolic process
BP	GO:0009960	endosperm development
BP	GO:0009567	double fertilization forming a zygote and endosperm
BP	GO:0009269	response to desiccation
CC	GO:0005811	lipid droplet
CC	GO:0012511	monolayer-surrounded lipid storage body
MF	GO:0120013	lipid transfer activity
MF	GO:0045735	nutrient reservoir activity

BP: Biological Process, CC: Cellular Component; MF: Molecular Function.

Supplementary Table 2: Monosaccharides molar ratio of EPF and EHF.

	Monosaccharide ratio				
	EPF				EHF
	Rha/GalA	Ara/Rha	Ara/Xyl	Gal/Rha	Xyl/Glc
Kallfu	2.46	0.57	0.54	0.65	0.87
Wenutram	2.51 (2%)	0.69 (22%)	0.68 (24%)	0.66 (2%)	0.86

Percentage showed the increase in the ratio based on the Kallfu ratio.

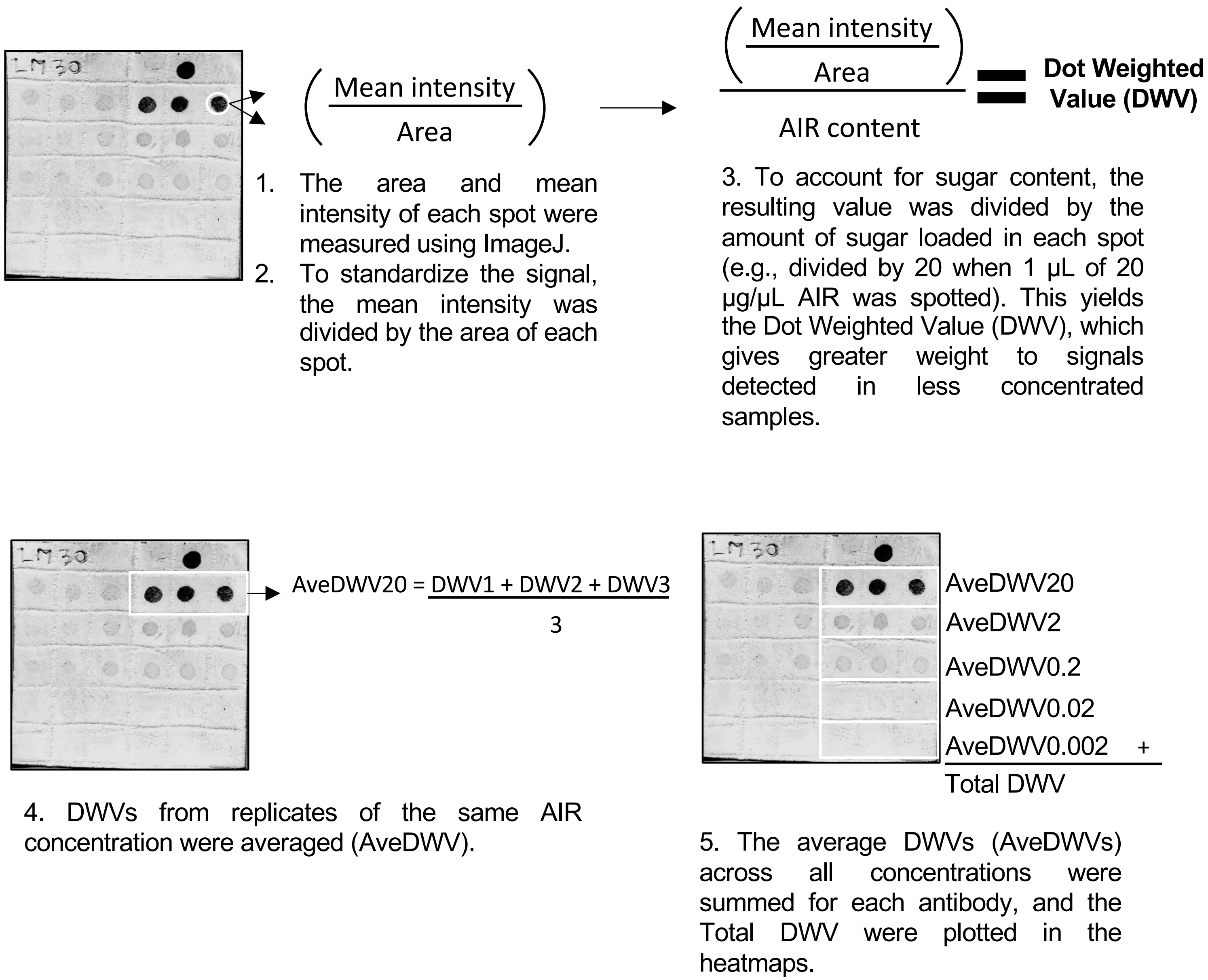

Supplementary Figure 1. Dot blot measurement and analysis. Example shown using AIR. **A.** Dot area and mean signal intensity were quantified using ImageJ. Signal intensity was standardized by the dot area. **B.** To weight the signal, intensity was normalized to the amount of AIR or sugar content in other fractions (e.g., EPF, EHF, and RG-I). This step gives more relevance to more diluted samples. **C.** Weighted values from replicates (DWV) were averaged. **D.** Finally, all averaged DWVs (AveDWVs) were summed to obtain the Total DWV, which was plotted as a heatmap (see Figure 2B and 4B).

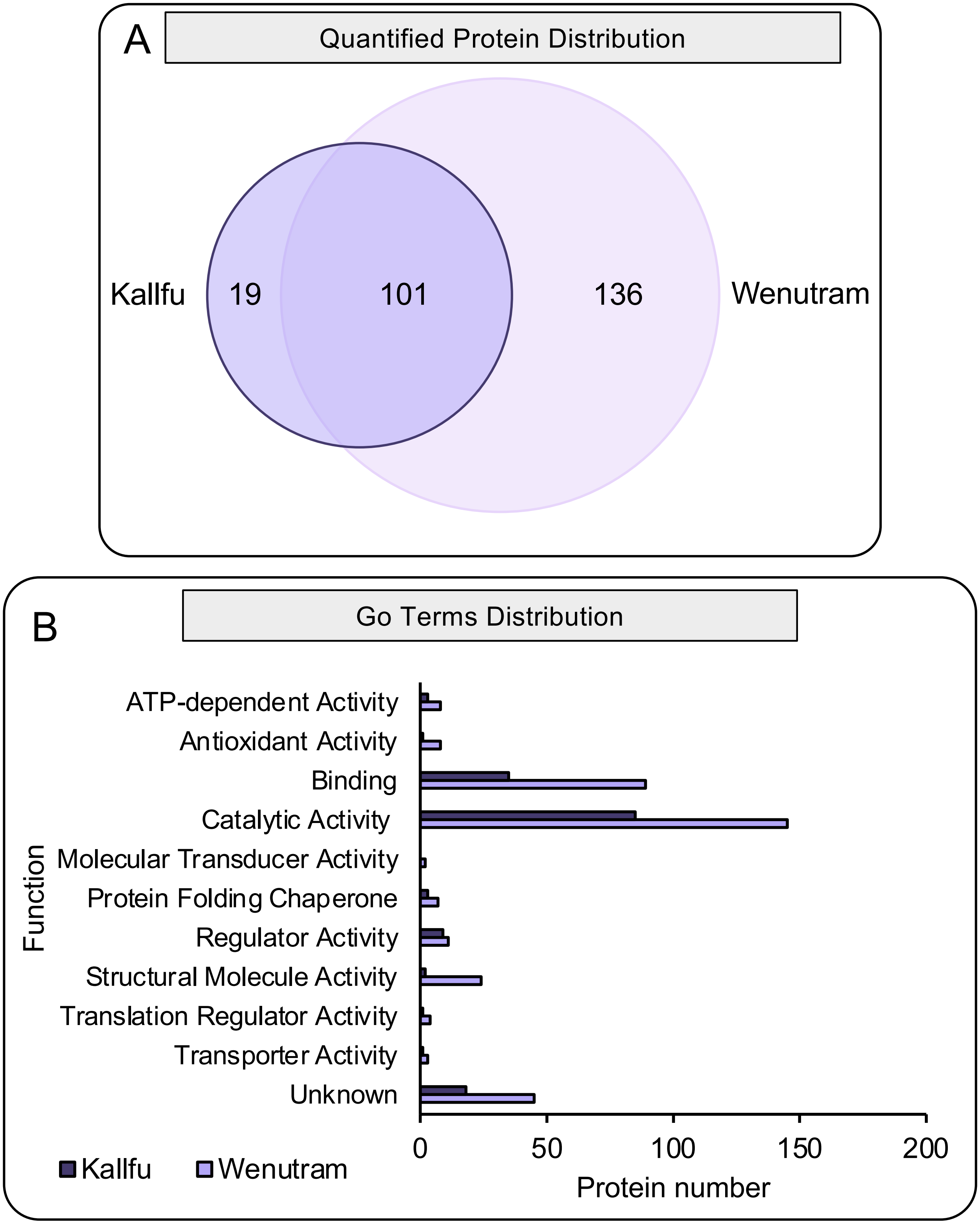

Supplementary Figure 2: Functions of the Identified Proteins in Kallfu and Wenutram Mucilage. A. Venn Diagram illustrating the distribution of proteins identified in Kallfu and Wenutram mucilage. **B.** The graph displays the GO terms associated with the proteins detected in Kallfu and Wenutram, allowing for a comparison of protein abundance between the two varieties.

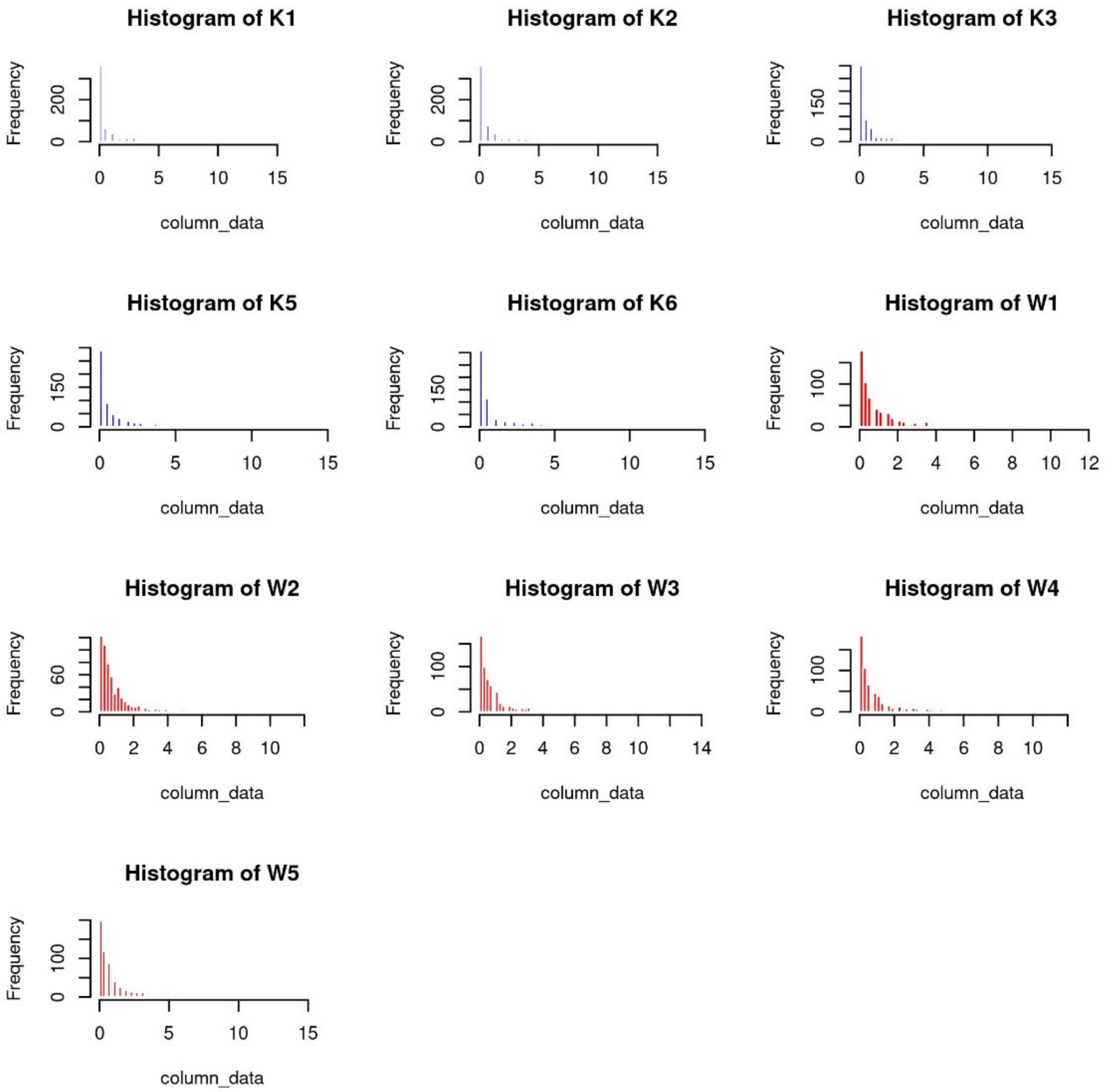

Supplementary Figure 3: Distribution of normalized peptide counts for flax cultivars. Blue and red bars represent Kallfu and Wenutram samples, respectively.

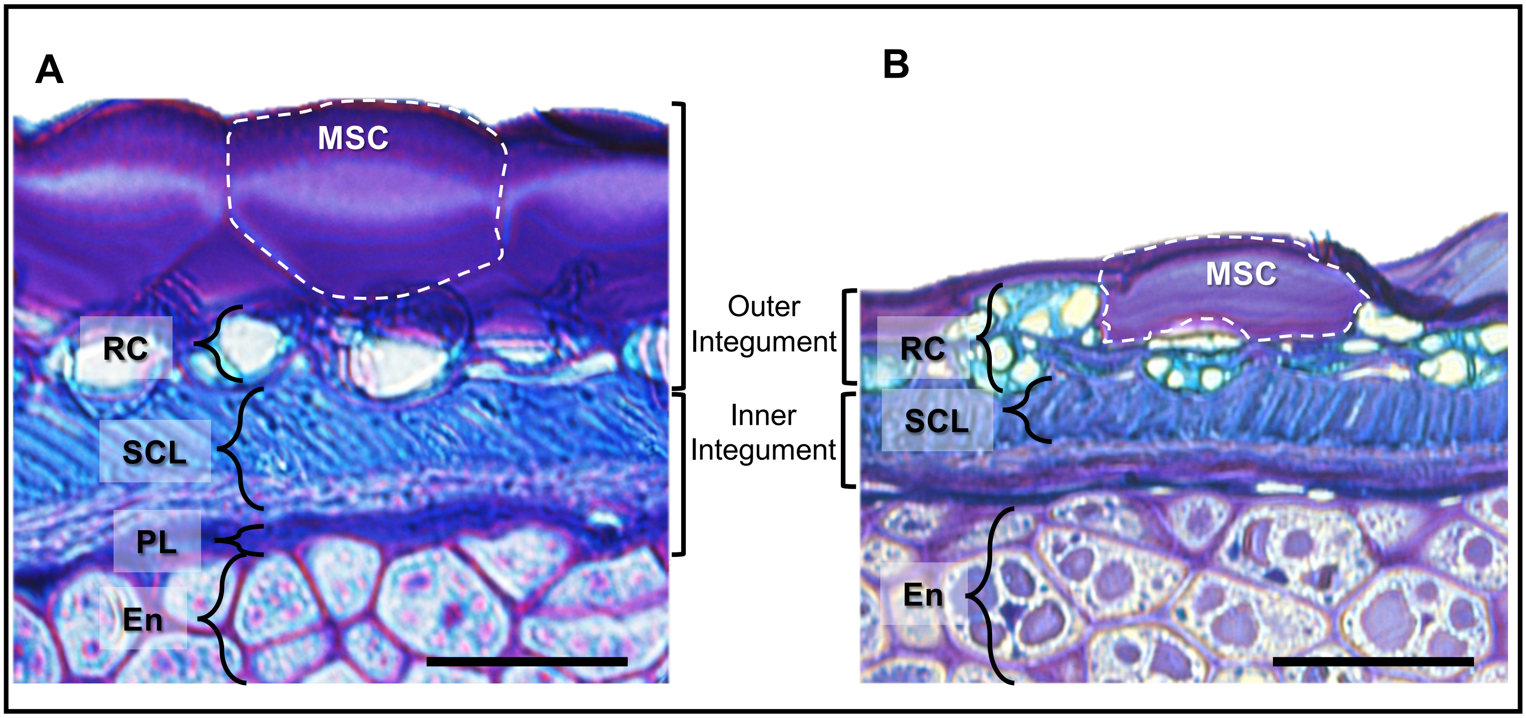

Supplementary Figure 4: Analysis of the seed coat structure of Kallfu and Wenutram. A, B. The figures show histological sections of the seed coats from Kallfu and Wenutram, respectively, stained with toluidine blue. The images reveal clear structural differences between the cultivars, with both the inner and outer integuments being more developed in Kallfu. This difference is particularly evident in the size of the mucilage secretory cells (MSC), which are significantly larger in Kallfu compared to Wenutram. MSC: Mucilage Secretory Cell. RC: Ring Cells. SCL: Sclerotic Cell Layer. PL: Pigment Layer. En: Endosperm. Bar= 50µm.

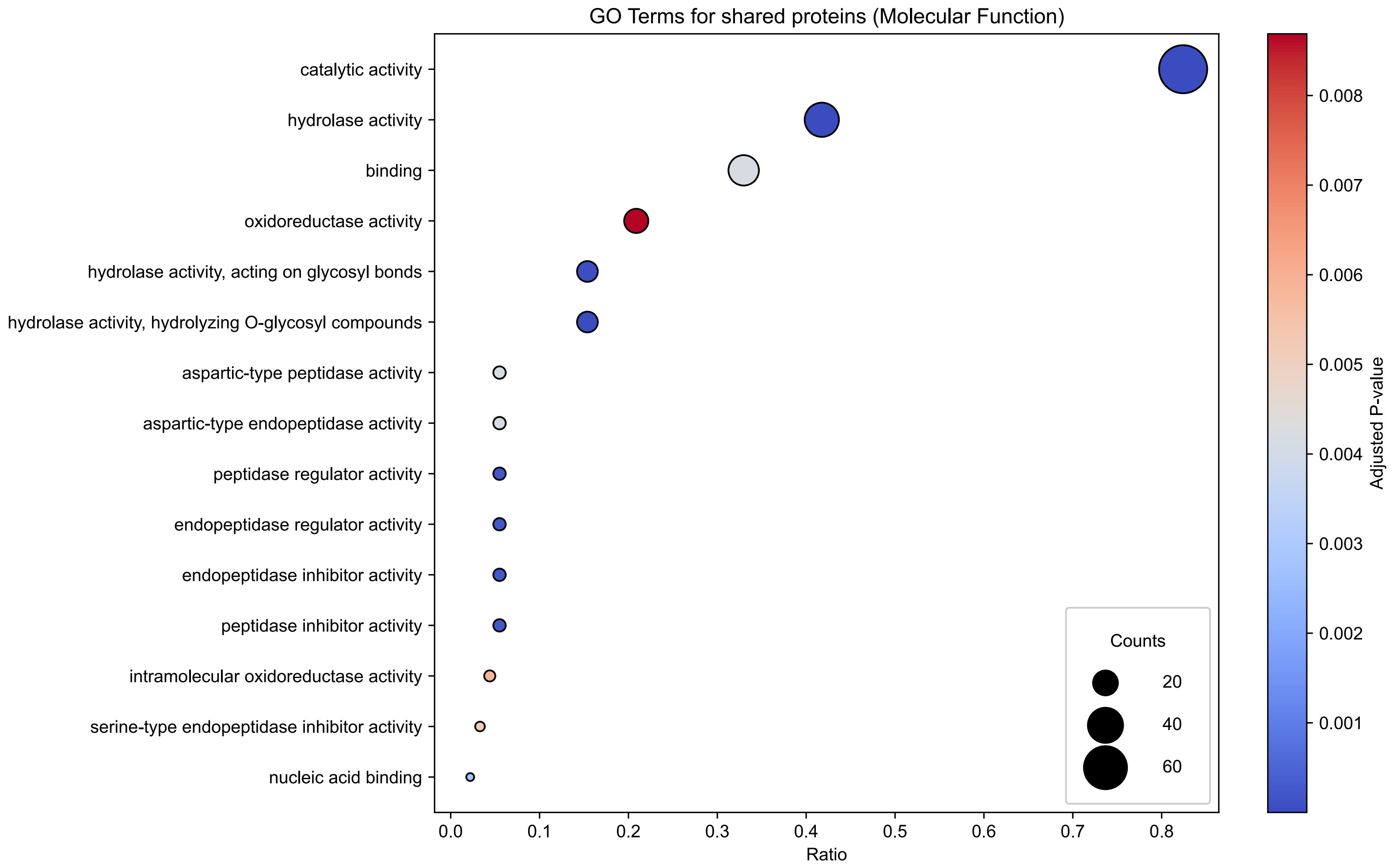

Supplementary Figure 5: Dot plot of enriched GO terms in both flax cultivars. Point size show peptide counts and color represent adjusted P value, with ascending P values from blue to red (right bar).

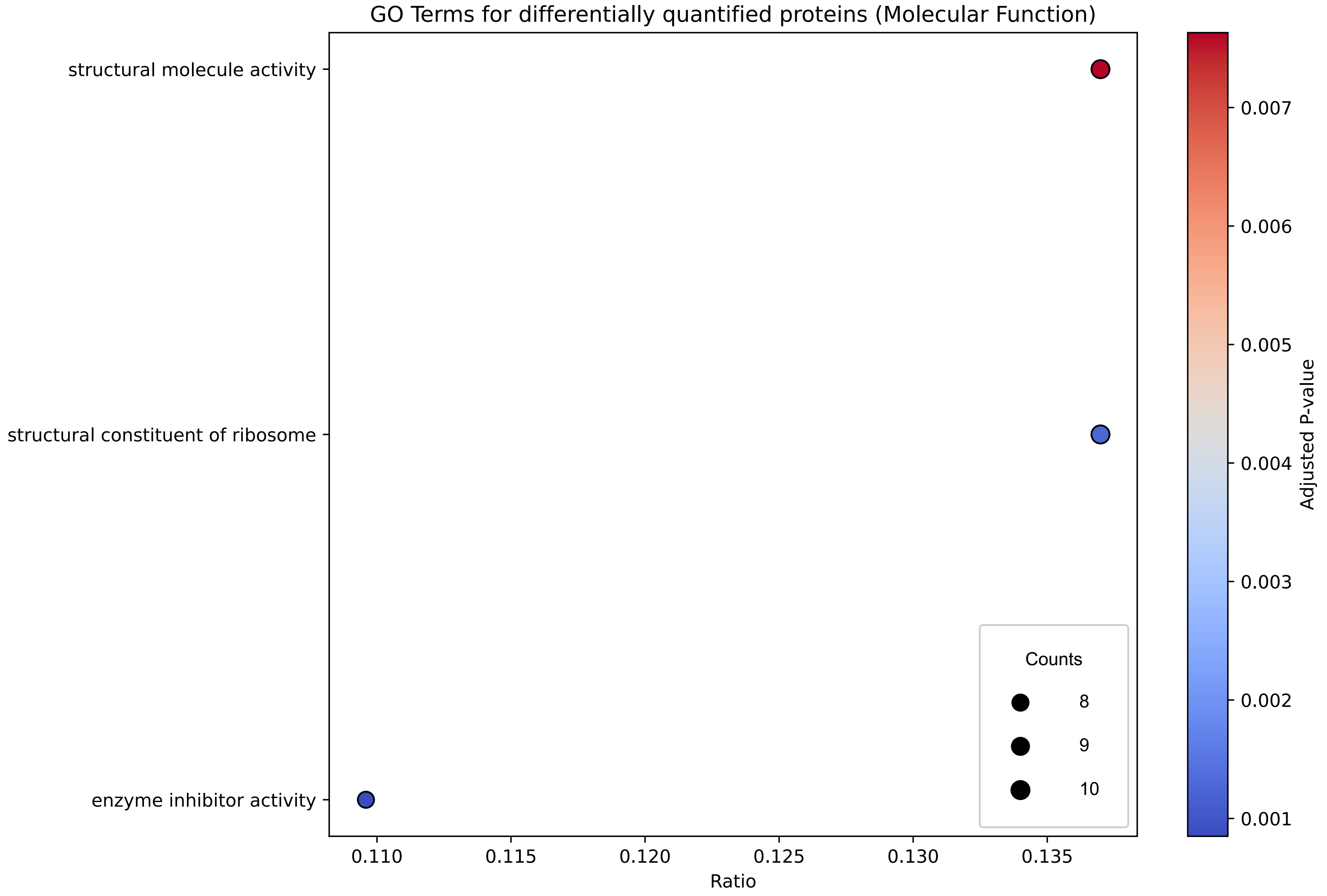

Supplementary Figure 6: Dot plot of enriched GO terms in Wenutram. Point size show peptide counts and color represent adjusted P value, with ascending P values from blue to red (right bar).

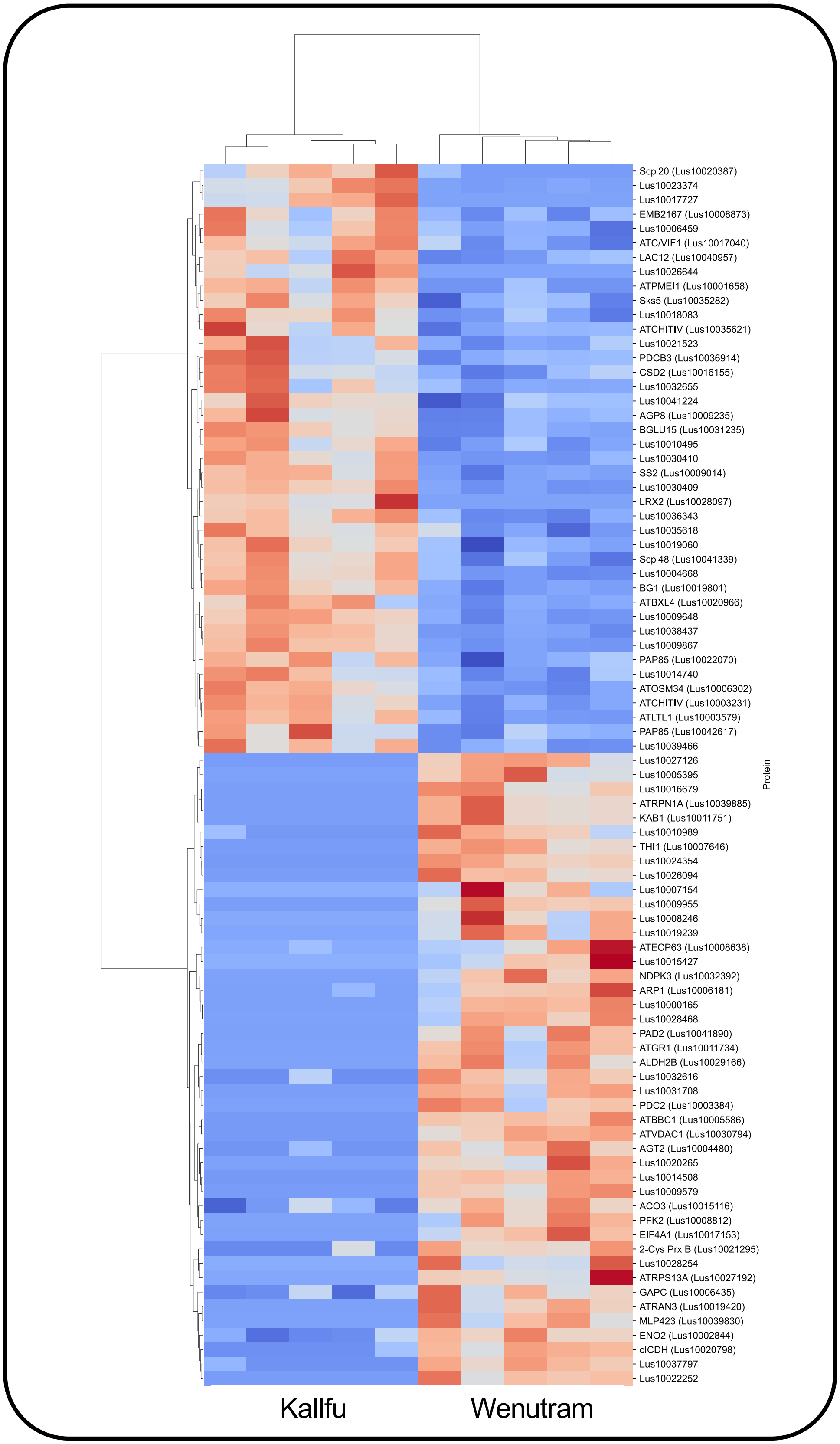

Supplementary Figure 7: Heatmap of Differentially Quantified Proteins between Kallfu and Wenutram. 85 protein groups were differentially quantified using a p-value < 0.01 Values are presented in z-score scale. The GO terms more represented were structural molecular activity, structural constituent of ribosome and enzyme inhibitor activity (Supplemental Figure 9).

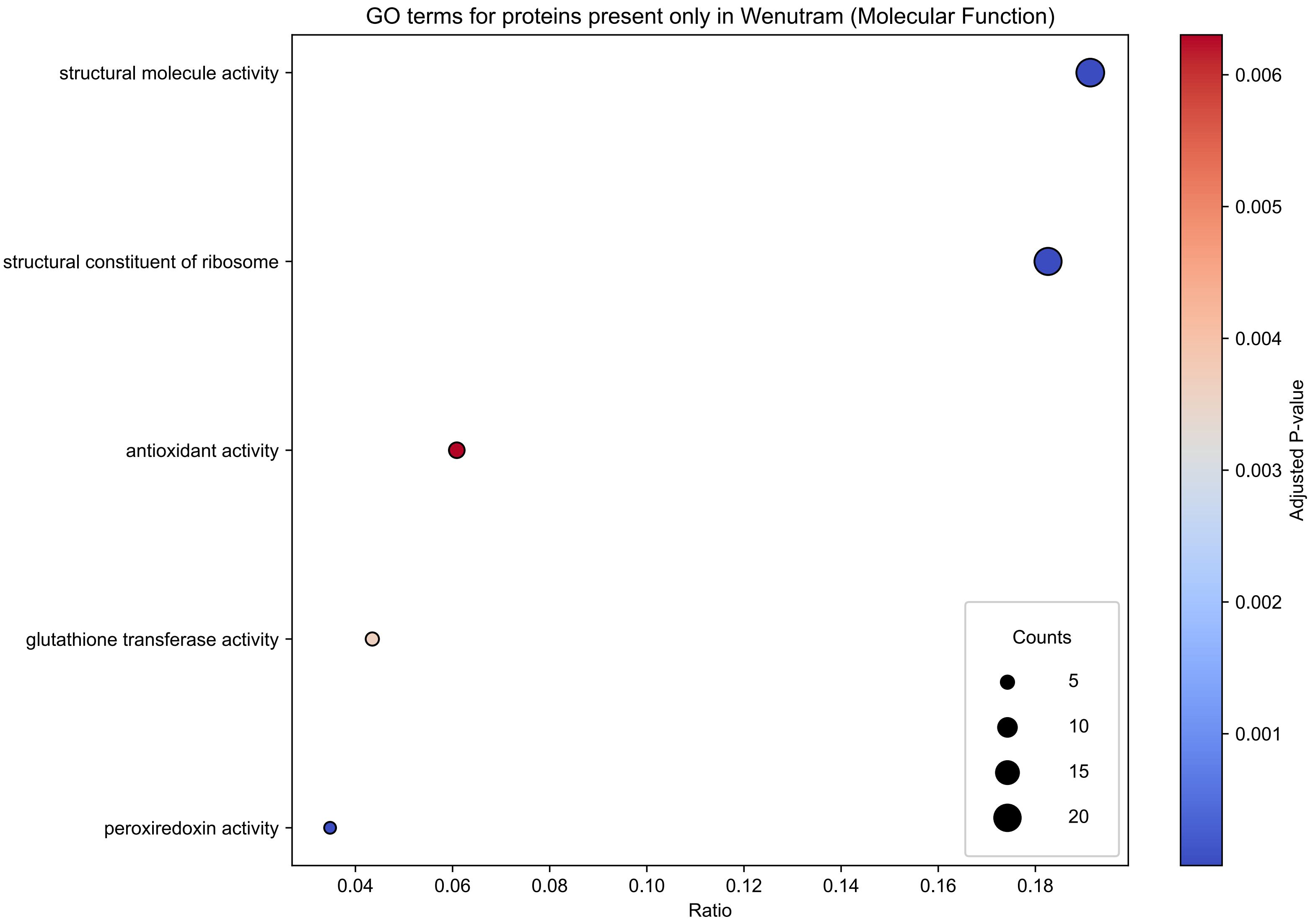

Supplementary Figure 8: Dot plot of enriched GO terms differentially quantified proteins. Point size show peptide counts and color represent adjusted P value, with ascending P values from blue to red (right bar).

A.

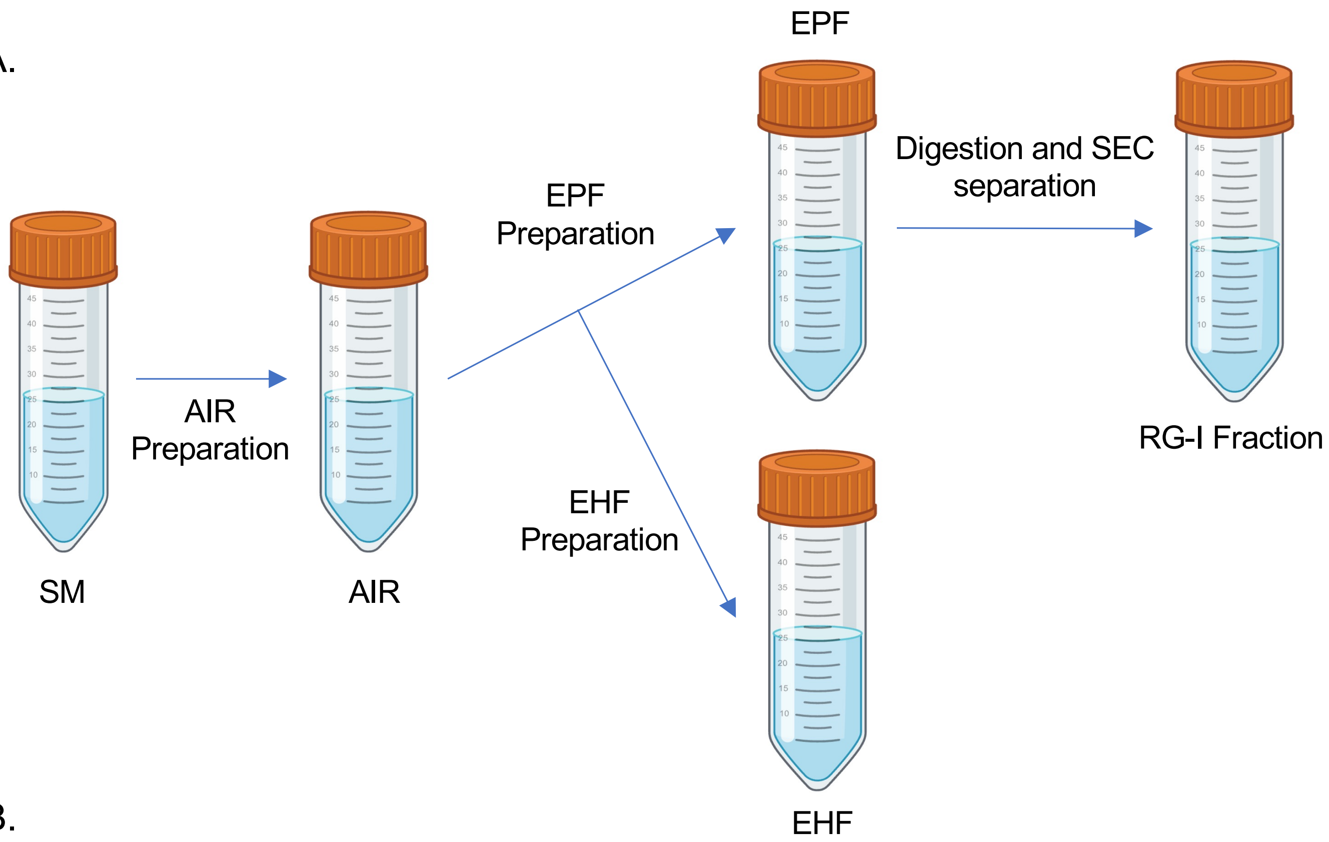

B.

Samples	Experiments		
	Protein Quantification	Sugar Analysis	Domain Detection
SM	X		
AIR		X (HPAEC-PAD)	X
EPF		X (HPAEC-PAD)	X
EHF		X (HPAEC-PAD)	X
RG-I fraction		X (Neutral Sugars)	X

Supplementary Figure 9: Sample preparation and final applications. A. Soluble mucilage (SM) was used to obtain alcohol-insoluble residue (AIR). From AIR, the EPF (pectin-enriched fraction) was isolated using chelators, and the supernatant was collected. The remaining pellet was used to isolate the EHF (hemicellulose-enriched fraction). RG-I was obtained by enzymatically digesting the EPF, followed by size-exclusion chromatography (SEC) separation. B. The different fractions were then used in various downstream experiments, as detailed in the accompanying table.

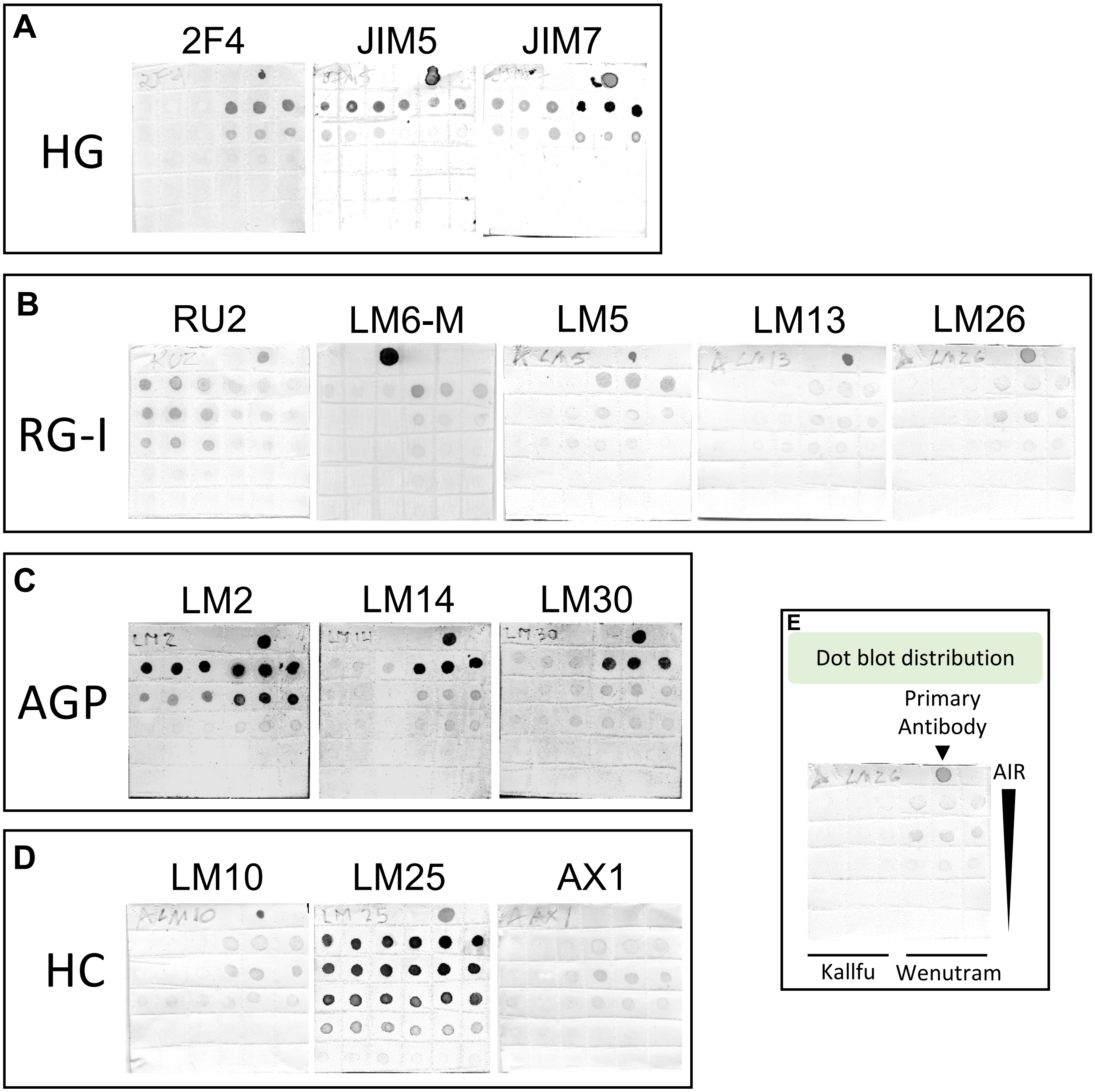

Supplementary Figure 10: Detection of specific CW epitopes in AIR. A. Detection of HG epitopes, **B.** Detection of RG-I, **C.** Detection of AGP, and **D.** Detection of HC (hemicellulose). **E.** Dot blot distribution, 1 µl of primary antibody is spotted as positive control. AIR concentration goes from more to less concentrated. Three replicates of Kallfu and Wenutram were spotted in serial dilution from a starting concentration of 20 µg/µl.

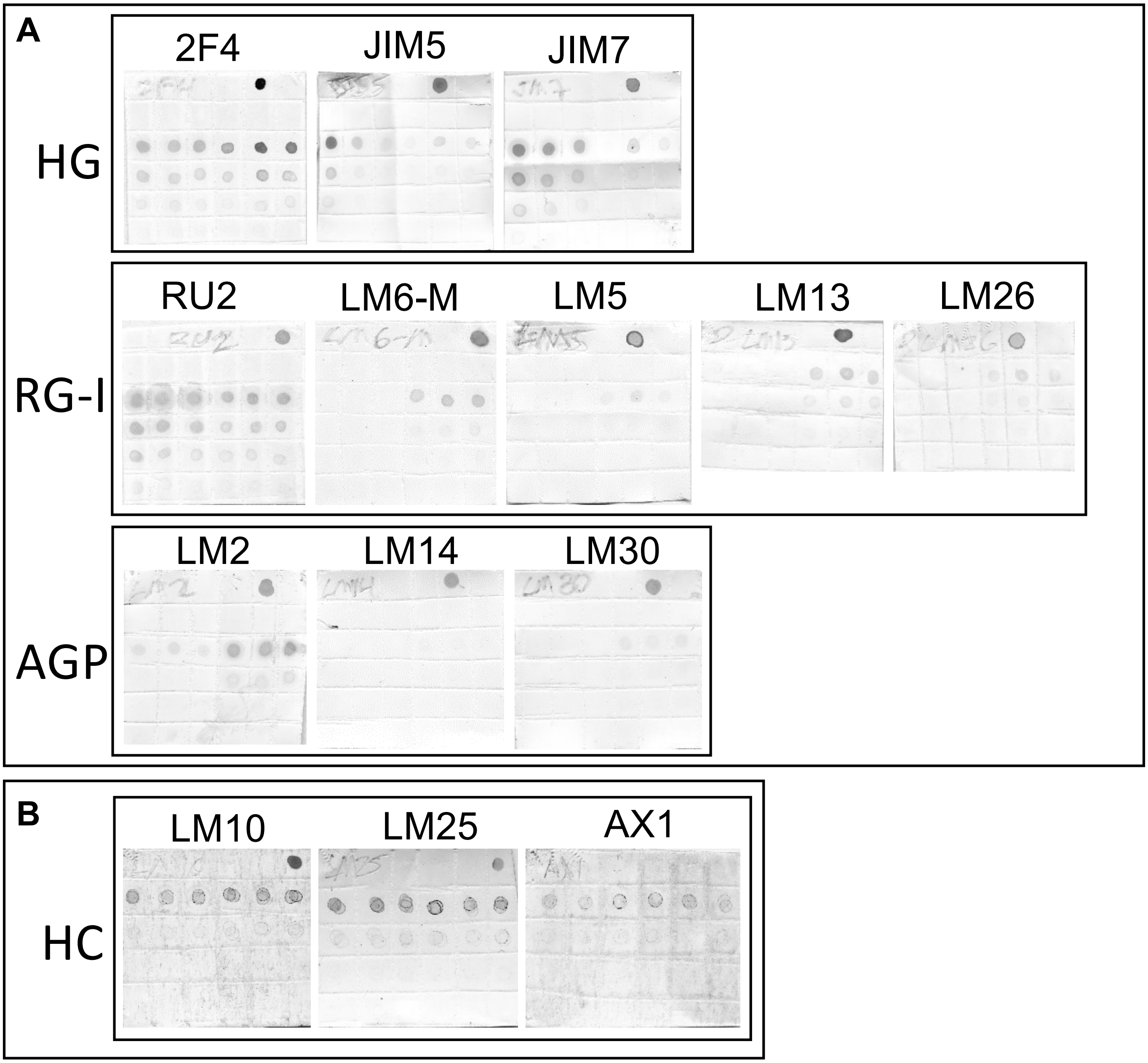

Supplementary Figure 11: Detection of specific CW epitopes in EPF and EHF. HG, RG-I, AGP, and HC (hemicellulose). **A.** Dot blot made with EPF. **B.** Dot blot made with EHF. EPF and EHF concentration go from more to less concentrated. Three replicates of Kallfu and Wenutram were spotted in serial dilution from a starting concentration of 3 µg/µl.

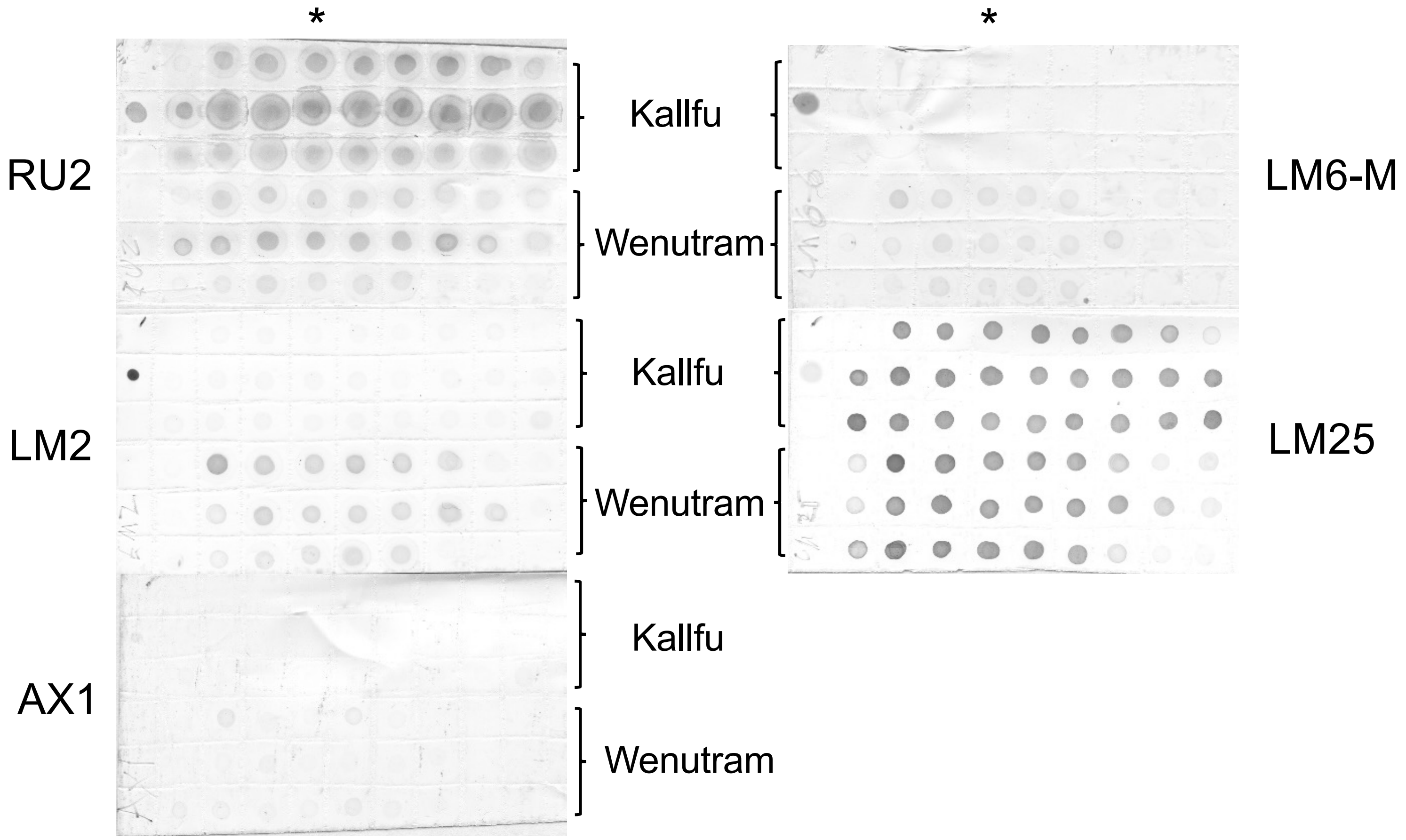

Supplementary Figure 12: Detection of specific CW epitopes in RG-I rich elution fraction. The elution with the higher GalA or neutral acid quantification is indicated by an asterisk. Three elution before and five elutions before the asterisk elution were analyzed by dot blot. RG-I was detected using INRA-RU2 antibody, AGP were detected using LM2, Arabinan was detected using the LM6-M and Xyloglucans were detected using LM25 antibody. Three replicates were used.
